# A foundation model for clinical-grade computational pathology and rare cancers detection

**DOI:** 10.1038/s41591-024-03141-0

**Published:** 2024-07-22

**Authors:** Eugene Vorontsov, Alican Bozkurt, Adam Casson, George Shaikovski, Michal Zelechowski, Kristen Severson, Eric Zimmermann, James Hall, Neil Tenenholtz, Nicolo Fusi, Ellen Yang, Philippe Mathieu, Alexander van Eck, Donghun Lee, Julian Viret, Eric Robert, Yi Kan Wang, Jeremy D. Kunz, Matthew C. H. Lee, Jan H. Bernhard, Ran A. Godrich, Gerard Oakley, Ewan Millar, Matthew Hanna, Hannah Wen, Juan A. Retamero, William A. Moye, Razik Yousfi, Christopher Kanan, David S. Klimstra, Brandon Rothrock, Siqi Liu, Thomas J. Fuchs

**Affiliations:** 1 Paige, New York, NY US; 2 Microsoft Research, Cambridge, MA US; 3 Memorial Sloan Kettering Cancer Center, New York, NY US; 4 NSW Health Pathology, St George Hospital, Sydney, New South Wales Australia; 5 University of Rochester, Rochester, NY US

**Keywords:** Diagnostic markers, Cancer screening, Pathology

## Abstract

The analysis of histopathology images with artificial intelligence aims to enable clinical decision support systems and precision medicine. The success of such applications depends on the ability to model the diverse patterns observed in pathology images. To this end, we present Virchow, the largest foundation model for computational pathology to date. In addition to the evaluation of biomarker prediction and cell identification, we demonstrate that a large foundation model enables pan-cancer detection, achieving 0.95 specimen-level area under the (receiver operating characteristic) curve across nine common and seven rare cancers. Furthermore, we show that with less training data, the pan-cancer detector built on Virchow can achieve similar performance to tissue-specific clinical-grade models in production and outperform them on some rare variants of cancer. Virchow’s performance gains highlight the value of a foundation model and open possibilities for many high-impact applications with limited amounts of labeled training data.

## Abstract (web summary)

Trained on 1.5 million whole-slide images from 100,000 patients, a pathology foundation model is shown to improve performance of specialized models in detection of rare cancers.

## Main

Pathologic analysis of tissue is essential for the diagnosis and treatment of cancer. Increasingly, the traditional histological preparations used for light microscopy examination are being replaced by their digital counterparts, also known as whole-slide images (WSIs), which enables the use of computational pathology^1–4^ to move from primarily academic proof points to routine tools in clinical practice. Computational pathology applies artificial intelligence (AI) to digitized WSIs to support the diagnosis, characterization and understanding of disease^5,6^. Initial work has focused on clinical decision support tools to enhance current workflows^7–14^, and in 2021 the first Food and Drug Administration-approved AI pathology system was launched^10^. However, given the incredible gains in performance of computer vision, a subfield of AI focused on images, more recent studies^15–19^ attempt to unlock new insights from routine WSIs and reveal undiscovered outcomes such as prognosis and therapeutic response^20^. If successful, such efforts would enhance the utility of hematoxylin and eosin (H&E)-stained WSIs and reduce reliance on specialized and often expensive immunohistochemistry (IHC) or genomic testing^21^.

A major factor in the performance gains of computer vision models has been the creation of large-scale deep neural networks, termed foundation models. Foundation models are trained on enormous datasets—orders of magnitude greater than any used historically for computational pathology—using a family of algorithms, referred to as self-supervised learning (for example, refs. ^22–26^), which do not require curated labels. Foundation models generate data representations, called embeddings, that can generalize well to diverse predictive tasks^27^. This offers a distinct advantage over current diagnostic-specific methods in computational pathology, which, limited to a subset of pathology images, are less likely to reflect the full spectrum of variations in tissue morphology and laboratory preparations necessary for adequate generalization in practice. The value of generalization from large datasets is even greater for applications with inadequate quantities of data to develop bespoke models, as is the case for the detection of uncommon or rare tumor types, as well as for less common diagnostic tasks such as the prediction of specific genomic alterations, clinical outcomes and therapeutic response. A successful pathology foundation model should capture a broad spectrum of patterns, including cellular morphology, tissue architecture, staining characteristics, nuclear morphology, mitotic figures, necrosis, inflammatory response, neovascularization and biomarker expression and therefore would be well-suited to predicting a wide variety of WSI characteristics. If trained with a sufficiently large quantity of digitized WSIs in the pathology domain, such a model could form the basis for clinically robust prediction of both common and rare cancers, as well as for other critical tasks such as subtyping of cancer, quantification of biomarkers, counting of cellular instances and events and the prediction of therapeutic response.

Foundation model performance crucially depends on dataset and model size, as demonstrated by scaling law results^28–30^. Modern foundation models in the natural image domain use millions of images (for example, ImageNet^31^, JFT-300M^32^ and LVD-142M^33^) to train models with hundreds of millions to billions of parameters (for example, vision transformers (ViTs)^34^). Despite the challenges in collecting large-scale datasets in the pathology domain, recent pioneering works have utilized datasets ranging from 30,000 to 400,000 WSIs to train foundation models ranging in size from 28 million to 307 million parameters^35–42^ (see Supplementary Note 1 for a detailed summary of recent models). These works demonstrate that image features produced with self-supervised learning of pathology images outperform image features trained on natural images and that performance improves with scale.

**Fig. 1:**
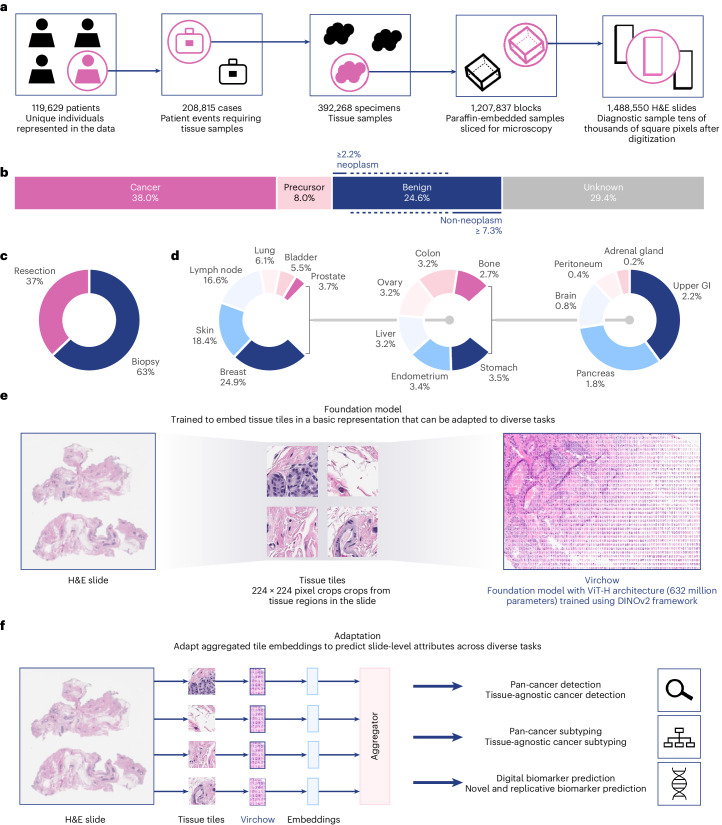
Overview of the study. The training dataset, training algorithm and application of Virchow, a foundation model for computational pathology. **a**, The training data can be described in terms of patients, cases, specimens, blocks or slides, as shown. **b**–**d**, The slide distribution as a function of cancer status (**b**), surgery (**c**) and tissue type (**d**). **e**, The dataflow during training requires processing the slide into tiles, which are then cropped into global and local views. **f**, Schematic of applications of the foundation model using an aggregator model to predict attributes at the slide level. GI, gastrointestinal.

Here, we present a million-image-scale pathology foundation model, Virchow, named in honor of Rudolf Virchow, who is regarded as the father of modern pathology^43,44^ and proposed the first theory of cellular pathology^45^. Virchow is trained on data from approximately 100,000 patients corresponding to approximately 1.5 million H&E stained WSIs acquired from Memorial Sloan Kettering Cancer Center (MSKCC), which is 4–10× more WSIs than in prior training datasets in pathology (detailed in Fig. 1a and ‘Million-scale training dataset’ in Methods). The training data are composed of cancerous and benign tissues, collected via biopsy (63%) and resection (37%), from 17 high-level tissues (Fig. 1b–d). Virchow, a 632 million parameter ViT model, is trained using the DINO v.2 algorithm^33^, a multiview student–teacher self-supervised algorithm (Fig. 1e; see ‘Virchow architecture and training’ in Methods for training details). DINO v.2 leverages global and local regions of tissue tiles to learn to produce embeddings of WSI tiles (Fig. 1e), which can be aggregated across slides and used to train a variety of downstream predictive tasks (Fig. 1f).

Motivated by highlighting the potential clinical impact of a pathology foundation model, we assess the performance of a pan-cancer model trained using the Virchow embeddings to predict specimen-level cancer across different tissues. Virchow embeddings outperform or match all baseline models on all tested cancer types, notably including rare cancers and out-of-distribution (OOD) data. Quantitative comparison to three specialized clinical-grade AI products demonstrates that the pan-cancer model performs nearly as well as the clinical products in general and outperforms them on some rare variants of cancers. To provide evidence for potential focus areas for future advances in computational pathology, qualitative analysis is also performed, characterizing the error patterns where the AI model fails to identify or falsely identifies cancerous cells. Motivated by simplifying clinical workflows, we evaluated the use of Virchow embeddings to train biomarker prediction, generally outperforming other models. Overall, our results provide evidence that large-scale foundation models can be the basis for robust results in a new frontier of computational pathology.

## Results

The Virchow model embeddings were evaluated on two categories of slide-level computational pathology applications: pan-cancer detection (‘Virchow enables pan-cancer detection’ and ‘Towards clinical-grade performance’) and biomarker prediction (‘Biomarker detection in routine imaging obviates additional testing’). These tasks require training a weakly supervised aggregator model to group tile embeddings to slide-level predictions. A series of tile-level linear probing benchmarks were also performed to directly assess the embeddings on individual tissue tiles (‘Tile-level benchmarks and qualitative analysis demonstrate generalizability’).

### Virchow enables pan-cancer detection

A key aim of our work was to develop a single model to detect cancer, including rare cancers (defined by the National Cancer Institute (NCI) as cancers with an annual incidence in the United States of fewer than 15 people per 100,000 (ref. ^46^)), across various tissues. The pan-cancer detection model infers the presence of cancer using Virchow embeddings as input. For evaluation, slides from MSKCC and slides submitted for consultation to MSKCC from numerous external sites globally are used. Stratified performance across nine common and seven rare cancer types is reported. Embeddings generated by Virchow, UNI^41^, Phikon^37^ and CTransPath^35^ are evaluated. Pan-cancer aggregators are trained using specimen-level labels, maintaining the same training protocol for all embeddings (see ‘Pan-cancer detection’ in Methods for data and training details).

**Fig. 2:**
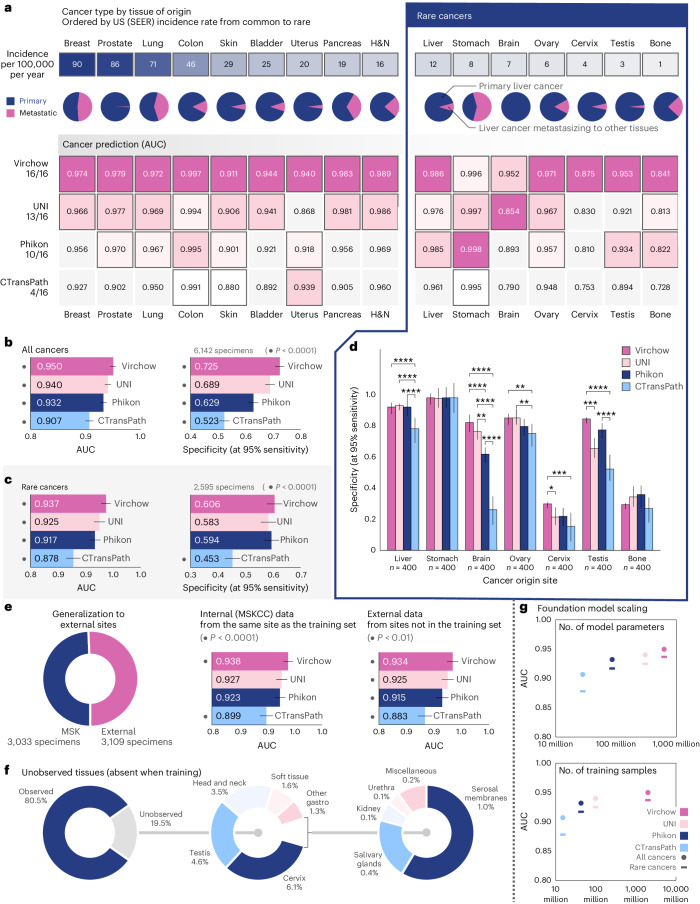
Virchow enables training a robust pan-cancer detector. Pan-cancer detection results. Detection is predicted at the specimen level using an aggregator network trained with Virchow, UNI, Phikon or CTransPath tile embeddings as input. **a**, Cancer detection performance (AUC) stratified by cancer type as determined by origin tissue. The incidence rate and proportion of metastasis of each cancer are shown. Virchow embeddings enable the best cancer detection performance across all cancer types, including rare cancers. For each cancer type, the AUC corresponding to the statistically significantly (*P* < 0.05) top-performing embeddings is highlighted in magenta. When more than one AUC is not gray, performance is ‘tied’ (no statistically significant difference). The foundation model used to produce tile embeddings for the aggregator is shown in the margin on the left, along with the number of cancer types for which the corresponding aggregator achieved (or tied for) the top AUC. All statistical significance (**a**–**e**) is computed using the pairwise DeLong’s test for AUC and Cochran’s *Q* test followed by McNemar’s test for specificity, both corrected for multiple comparisons with Holm’s method. **b**,**c**, Cancer detection performance summarized for all cancers (**b**) and for rare cancers (**c**). Error bars (**b**–**e**) show the two-sided 95% confidence interval computed with DeLong’s method for AUC and Wilson’s method for specificity; the • denotes the differences that are statistically significant from the rest (*P* < 0.0001). **d**, Sensitivity at 95% specificity for rare cancer detection (*P < 0.05, **P < 0.01, ***P < 0.001, ****P < 0.0001). **e**, Virchow-based cancer detection generalizes well to data from external institutions that were not represented in the training set; all aggregators and Virchow were trained only on data from MSKCC. Only half of the specimens in the pan-cancer testing set are from MSKCC. **f**, One-fifth of the specimens used for pan-cancer model evaluation contained tissues that were not observed in the training sets of Virchow or the pan-cancer aggregators. **g**, Cancer detection performance scales with the size of the underlying foundation model and the number of training samples (tiles) used to train it. H&N, head and neck.

Virchow embeddings yielded the best cancer detection performance on all cancer types (Fig. 2a). Pan-cancer detection using UNI embeddings achieved statistically similar performance (*P* < 0.05) for eight of the nine common cancer types and five of the seven rare cancer types; nevertheless, in all but one case, the specific area under (the receiver operating characteristic) curve (AUC) score was lower. Overall the pan-cancer model achieved an AUC of 0.950 with Virchow embeddings, 0.940 with UNI embeddings, 0.932 with Phikon embeddings and 0.907 with CTransPath embeddings (Fig. 2b; all significantly different with *P* < 0.0001). See Extended Data Fig. 3 for more detailed AUC and specificity metrics, stratified by cancer type.

Rare cancer detection performance is particularly noteworthy. Compared to the aforementioned AUC of 0.950 overall, Virchow embeddings yielded an AUC of 0.937 on rare cancers (Fig. 2c), demonstrating generalization to rare data. Performance across the individual rare cancers was, however, non-uniform, with detection of cervical and bone cancers proving more challenging (AUC < 0.9) irrespective of the embeddings used (Fig. 2a,d). Virchow embeddings improved cervix detection to 0.875 AUC compared with 0.830, 0.810 or 0.753 when using UNI, Phikon or CTransPath embeddings, respectively. Similarly, Virchow embeddings yielded 0.841 AUC for bone cancer detection, compared to 0.813, 0.822 and 0.728 with UNI, Phikon and CTransPath, respectively. At 95% sensitivity, we show that a pan-cancer detection model using Virchow embeddings can achieve 72.5% specificity, compared to 68.9%, 62.9% or 52.3% using UNI, Phikon or CTransPath embeddings, respectively, trained on less data (Fig. 2b).

The robustness of Virchow embeddings to data sampled from a different population than the training set (OOD data) is evaluated directly with data from institutions other than MSKCC (both Virchow and the pan-cancer aggregator were trained only on data from MSKCC) and indirectly by including data from tissues which were not observed during training (Fig. 2e,f). As AUC measures cannot be exactly compared across different data subsets (due to different positive to negative sample ratios), we report AUC for all pan-cancer models on all data or rare cancers (Fig. 2b), as well as on internal or external data (Fig. 2e), and demonstrate that the AUC differences across models remain consistent in each subpopulation. This demonstrates that Virchow embeddings generalize well to new or rare data and outperform the others consistently. Although AUC cannot be exactly compared across data subsets, we can observe that all models achieve a similar AUC on both internal and external data, suggesting that they generalize well as external data can be challenging because it is submitted to MSKCC for consultation. Furthermore, cervix, testis and head and neck (H&N) are tissues not seen during training, and Virchow embeddings still outperform competing models. Overall, pan-cancer detection generalizes across cancer types, including rare cancers, as well as on OOD data when using foundation model embeddings.

The comparison of pan-cancer performance based on different foundation model embeddings reveals that performance scales with the size of the foundation model and the size of the training data (Fig. 2g). Cancer detection was found to scale approximately logarithmically with the number of model parameters (Fig. 2g, top); although performance scaled with the number of training tile samples, the trend (Fig. 2g, bottom) suggests diminishing returns. Although the training datasets, model architectures and optimization strategies differ across Virchow, UNI, Phikon and CTransPath, there are enough similarities to motivate the scaling analysis. All models are transformer-based: CTransPath uses a Swin transformer^47^, and the rest use ViTs^34^ of different sizes. Phikon was trained using the iBOT algorithm^23^, and both Virchow and UNI were trained using the DINO v.2 algorithm^33^ with similar hyperparameters. iBOT and DINO v.2 are related approaches as the latter builds on the masked image modeling proposal of the former. CTransPath is differentiated in terms of training algorithm as it used a contrastive learning algorithm based on MoCov3 (ref. ^48^). To learn about the effect of dataset size independent of model size, we direct the reader to the study in ref. ^41^.

### Toward clinical-grade performance

**Fig. 3:**
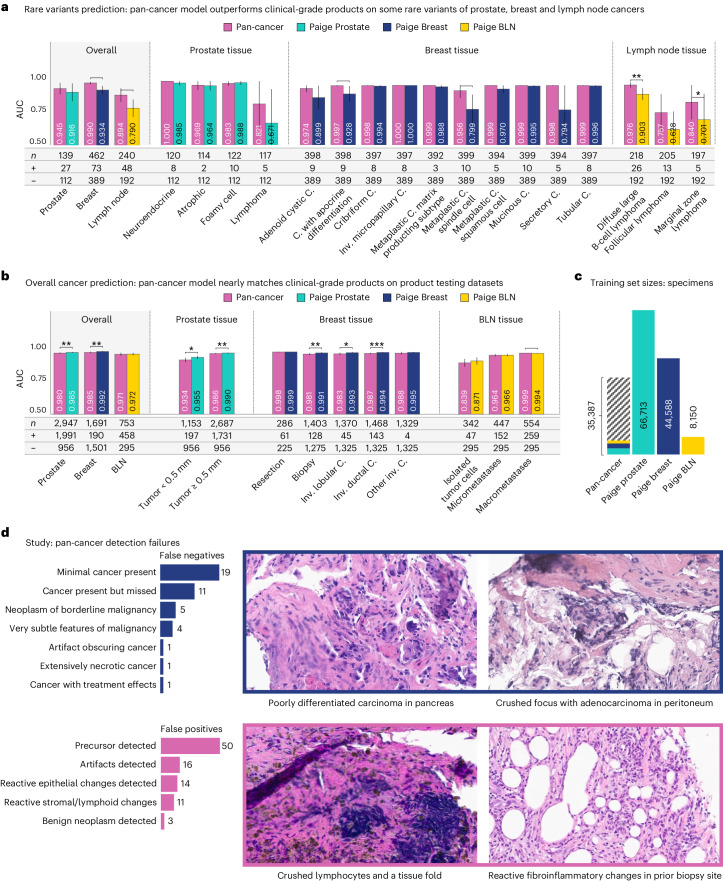
Pan-cancer detection approaches and sometimes surpasses clinical product performance, using less data. **a**,**b**, Performance as measured by AUC of three clinical products compared to the pan-cancer model trained on Virchow embeddings, on the rare variant (**a**) and product testing datasets (**b**). The pan-cancer detector, trained on Virchow foundation model embeddings, achieves similar performance to clinical-grade products in general and outperforms them on rare variants of cancers. **c**, The pan-cancer detector was trained on fewer labeled specimens than the Prostate, Breast and BLN clinical models, including a small fraction of the prostate (teal), breast (blue) and BLN (yellow) tissue specimens that these clinical models were respectively trained on. **d**, A categorization of failure models of the pan-cancer model and four canonical examples of the primary types of failures. In all panels, * is used to indicate pairwise statistical significance (*P < 0.05, **P < 0.01, ***P < 0.001, ****P < 0.0001; pairwise DeLong’s test). Error bars denote the two-sided 95% confidence interval, estimated with DeLong’s method. C., carcinoma. Inv., invasive.

A promise of foundation models is improved generalization; however, this claim is difficult to verify without access to rigorously trained and tested tissue-specific specialist models. To this end, we conducted a comparative analysis between the Virchow-based pan-cancer detection model and specialist commercial models, specifically Paige Prostate, Paige Breast and Paige Breast Lymph Node (BLN). The comparison focuses on the AUC for cancer detection, specifically for prostate cancer, invasive breast cancer and metastases of breast cancer in lymph nodes. These commercial models were trained using multiple-instance weakly supervised learning as described in refs. ^14,49^ specifically for cancer detection. The evaluation was performed in two settings: (1) product testing datasets and (2) rare cancer variant datasets in the respective tissues (Fig. 3b–d).

The Virchow-based pan-cancer detection model, trained on cancers across numerous tissues, performs nearly as well as the prostate, breast and BLN clinical specialist models (Fig. 3c) while outperforming them on many rare variants of cancers (Fig. 3d). It is important to note that the pan-cancer training set did not benefit from the same refinement as the product training sets, such as enrichment for subpopulations and label quality control. Furthermore, the pan-cancer model was trained on fewer tissue-specific specimens than the clinical models (Fig. 3 and Extended Data Fig. 4). Concretely, Paige Prostate was trained on 66,713 blocks, Paige Breast was trained on 44,588 specimens and BLN on 8150 specimens, whereas pan-cancer (using Virchow embeddings) was trained on only 35,387 groups of slides (blocks or specimens) in total, of which 2,829 are prostate, 1,626 are breast and 1,441 are lymph node. The pan-cancer model achieves an AUC of 0.980, 0.985 and 0.971 on prostate, breast and BLN, respectively. This performance approaches that of commercial models; however, it is still surpassed by the Food and Drug Administration-approved Paige Prostate model (0.980 versus 0.995 AUC, *P* < 0.05) and the Paige Breast model (0.985 versus 0.992 AUC, *P* < 0.01). On the other hand, it is statistically significantly better at detecting macrometastases than Paige BLN (0.999 versus 0.994 AUC, *P* < 0.05). Furthermore, there is no statistically significant difference (*P* < 0.05) in the other BLN comparisons or some of the stratified breast cancer comparisons (Fig. 3c).

In addition to approaching the specialist models in terms of overall AUCs, the pan-cancer model matches or outperforms these models on rare variants of cancers, as shown in Fig. 3d. In prostate and lymph node tissues, the pan-cancer model is capable of detecting lymphoma. This is particularly noteworthy because none of the models were trained in hematolymphoid malignancies. Owing to their different lineage (carcinomas originate from epithelial cells, whereas lymphomas arise from lymphoid tissue) their morphologic appearance tends to be quite different. In two of the four lymphoma variants, the pan-cancer model outperforms the specialized model. Improved detection of diffused large B-cell lymphoma is noteworthy as this variant is particularly aggressive. In breast tissue, the pan-cancer model outperforms the Paige Breast model overall and especially on some rare histological variants, including adenoid cystic carcinoma, carcinoma with apocrine differentiation (*P* < 0.05), metaplastic carcinoma spindle cell (*P* < 0.01), metaplastic carcinoma squamous cell and the exceptionally unusual secretory carcinoma. We note that due to the rarity of these variants of cancers, rare variants prediction lacks the statistical power of the product datasets.

To comprehend the error patterns of the pan-cancer model across various tissues, a pathologist examined the error cases within a curated set of evaluation WSIs (see ‘Pan-tissue product benchmark’ in the section ‘Clinical evaluation datasets’ in Methods). The operating point for each tissue was selected to achieve approximately 95% sensitivity and 85% specificity on a tuning dataset. These error patterns were documented using free text first, which was subsequently categorized to provide a comprehensive summary. We posit that these patterns could be beneficial to similar cancer detection studies, providing valuable insights for the enhancement of future foundational models and clinical AI applications. The false positive and false negative patterns were analyzed separately, as depicted in Fig. 3e.

Upon analysis of the false positive and false negative cases, it was discerned that a substantial proportion could be attributed to specific findings. Histological preparations that contained only small tumoral foci constituted the majority (45.2%) of the false negatives. Certain neoplasms, undetected as cancer (11.9%), were of borderline malignant potential, such as gastrointestinal stromal tumors or borderline serous neoplasm of the ovary. Others (9.5%), such as low-grade astrocytoma, exhibited only very subtle histologic features of malignancy. Treatment effects, extensive necrosis and tissue artifacts obscuring the cancer accounted for a few false negatives. In 11 cases (26.2%), there was more than minimal cancer within the specimen, and the negative result of the model could not be explained.

The majority of the false positive cases fell into two categories. Precursor lesions in specimens lacking invasive cancer constituted most (53.2%) of the false positives. These were found most frequently in the bladder, breast, cervix, skin (squamous dysplasia) and esophagus. Most detected precursors exhibited high-grade dysplasia, with cytologic features resembling those of invasive carcinoma, although some foci of low-grade dysplasia were also detected in the gastroesophageal junction and skin. The second most common (17.0%) cause of false positive results was tissue artifacts, especially crush artifacts (in which non-neoplastic cells are physically crushed during sample preparation, resulting in a characteristic streaming effect of the nuclei), tissue folds and out-of-focus regions. Reactive alterations within the stroma or lymphoid components, constituting 14.9%, and in non-neoplastic epithelial tissue, representing 11.7%, were also responsible for false positive results. A number of these findings, such as biopsy site changes, reactive epithelial atypia, glandular atrophy and acellular stromal mucin, are well-recognized malignant mimics that challenge pathologists as well. Three cases (3.2%) were benign neoplasms misidentified as cancer. These included benign gastrointestinal stromal tumors, hepatic angiomyolipomas and serous cystadenomas of the pancreas.

### Biomarker detection in routine imaging obviates additional testing

**Fig. 4:**
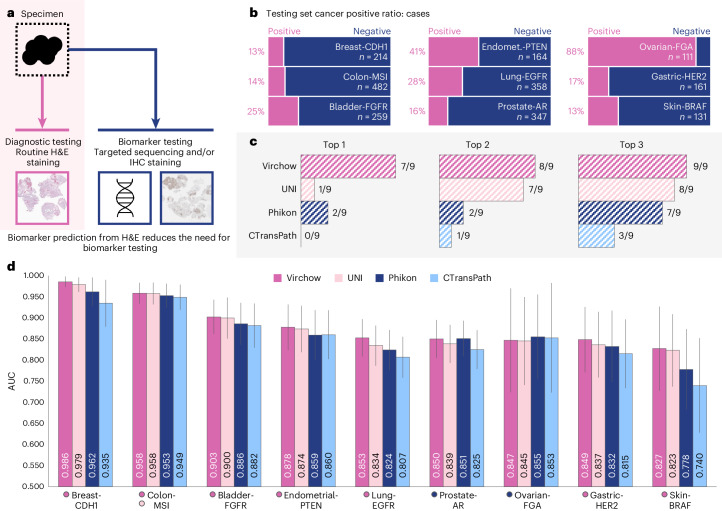
Biomarker prediction results. **a**, Virchow embeddings help predict biomarkers directly from H& E slides, reducing the need for targeted sequencing or IHC staining. **b**, The fraction of positive cases in each biomarker testing dataset. **c**, The number of biomarkers on which using Virchow, UNI, Phikon or CTransPath embeddings to train an aggregator produced an AUC in the top *x*. This ranking does not consider statistical significance across models for each biomarker due to low statistical power; instead, it relies on considering the ranking across many biomarkers. **d**, Biomarker detection performance as measured by AUC using aggregator networks trained on embeddings from Virchow, Phikon or CTransPath. For each prediction task, the top scoring embeddings are marked with a colored circle next to the biomarker label, below the plot (this corresponds to the top-1 ranking in **c**). The error bars denote the two-sided 95% confidence interval computed from 1,000 bootstrapping iterations. Endomet., endometrial.

The prediction of biomarkers from standard H&E stained images can reduce the reliance on testing using additional methods and the associated substantial delays in returning results to patients (Fig. 4a). The status of a biomarker in a specimen is predicted using an aggregator network with the foundation model embeddings as input. These biomarkers play a crucial role in the diagnosis and treatment of various cancers, and each is described in further detail in ‘Biomarker detection’ in Methods (see also Supplementary Table 3.1 and Fig. 4b). The biomarker detection datasets consist of WSIs from the histological sections matching the blocks used for DNA extraction and MSK-integrated mutation profiling of actionable targets (MSK-IMPACT) sequencing^50^, the latter of which was analyzed to determine the status of genetic alterations and establish a binary label indicating the presence or absence of the variants: that is, the biomarker (Fig. 4a). Similar to the pan-cancer evaluation, the publicly available UNI^41^, Phikon^37^ and CTransPath^35^ models are used as baseline models for comparisons.

We note that the biomarker prediction results lacked sufficient statistical power to assess statistically significant differences across models; instead, we conclude relative model performance from evaluating many different biomarker predictions. In our comparative analysis shown in Fig. 4c, Virchow embeddings demonstrated superior performance in seven of the nine evaluated digital biomarkers, achieving AUC scores that exceeded those of the nearest baseline foundation models. This performance underscores the robustness of Virchow embeddings across a diverse range of biomarkers. Even in the categories of prostate–androgen receptor (AR) and ovarian–fraction of genome altered (FGA), where Virchow did not secure the top position, it remained a strong contender, with AUCs of 0.849 and 0.847, respectively. These findings underscore the potential of Virchow embeddings to accurately represent H&E histologic phenotypes, offering predictive insights into biomarkers that are traditionally identified through DNA extraction and MSK-IMPACT sequencing.

### Tile-level benchmarks and qualitative analysis demonstrate generalizability

To directly evaluate tile-level embeddings without the confounder of training an aggregator network, we evaluated Virchow performance on a set of tile-level benchmarks by linear probing. Linear probe evaluation aims to gauge the quality and separability of representations learned by a self-supervised model. We compare Virchow embeddings to baseline model embeddings by applying the same linear probing protocol for each model, using the same training, validation and testing data splits (see ‘Tile-level benchmarking’ in Methods for further details). The analysis is performed both on public datasets and on an internal MSKCC pan-cancer dataset.

The internal multitissue dataset for pan-cancer detection at the tile level (referred to as PanMSK) is an in-distribution benchmark, as it is composed of annotations on a held out set of patients across the entire diverse set of tissue groups selected for training (Fig. 1d). The public datasets are OOD benchmarks and are described in the ‘Tile-level benchmarking’ section in Methods. In addition to UNI^41^, Phikon^37^ and CTransPath^35^, DINO_*p*=8_ (ref. ^39^) (49 million parameter model trained using The Cancer Genome Atlas (TCGA) and an internal dataset), PLIP^51^ (87 million parameter model trained using pathology image-text pairs) and NatImg^33^ (1.1 billion parameter model trained on 142 million natural images) are evaluated.

**Fig. 5:**
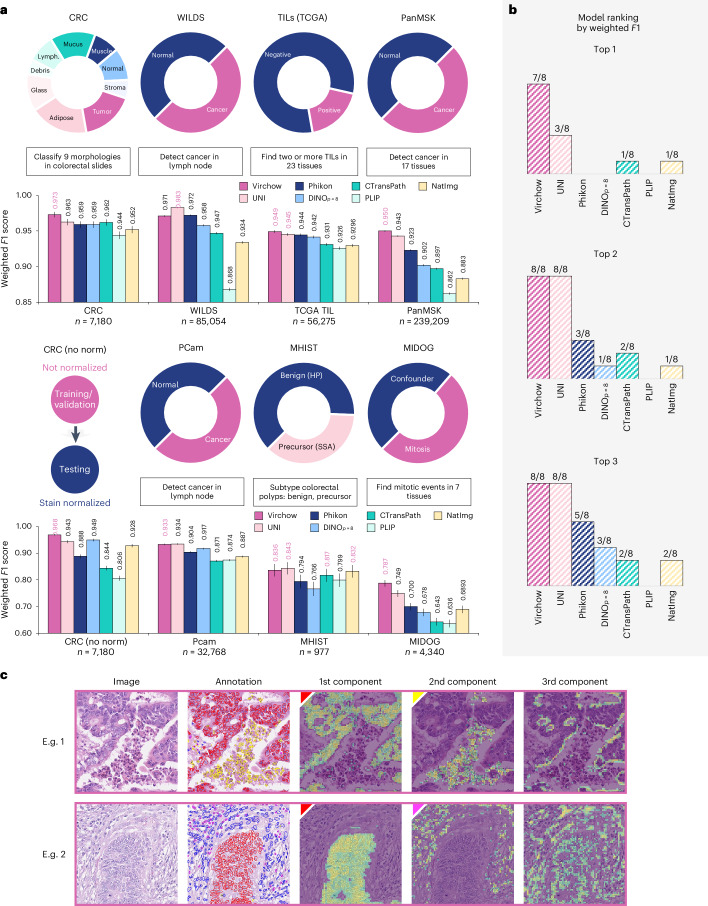
A summary of tile-level linear probing. **a**, A description of each tile-level benchmark (top) along with the corresponding results for the embeddings of different foundation models (bottom). For each task, the top result is bolded and highlighted in magenta. Multiple results are highlighted when there is no statistically significant difference between them (*P* < 0.05; McNemar’s test). Error bars denote two-sided 95% confidence intervals computed using 1,000 bootstrapping iterations. **b**, The number of tasks in which each model scored in the top *x*. Models can tie for a rank depending on statistical significance (*P* < 0.05). **c**, Virchow embedding features learn meaningful structures. Cells in the CoNSeP dataset highlighted by embedding principal components: malignant epithelium (red), miscellaneous (yellow) and inflammatory (magenta).

As shown in Fig. 5a,c, Virchow embeddings match or surpass the performance of other embeddings in seven of the eight benchmark tasks (Fig. 5a,b; see Supplementary Table 4.2 for additional metrics). The closest competing models are UNI and Phikon, with UNI scoring in the top 1 three times and in the top 2 for all tasks and Phikon scoring in among the top 2 three times. Virchow demonstrates strong OOD performance as measured by the WILDS and ‘CRC (no norm)’ tasks. The WILDS test data is sourced from a hospital that is not encountered in the training set. The ‘CRC (no norm)’ task introduces a distribution shift from the stain-normalized training set by avoiding stain normalization on the testing set. Without normalization, Virchow’s performance declines by only −0.005 in weighted *F*1 score, indicating robustness to variations in data preprocessing.

To qualitatively evaluate whether the embeddings learned by Virchow tend to separate the image into semantically meaningful clusters of features, we performed an unsupervised feature analysis similar to the procedure in ref. ^33^ using the CoNSeP dataset^52^, which contains H&E stained slides of colorectal adenocarcinoma (detailed under ‘Qualitative feature analysis’ in Methods).

We observe approximate semantic segmentation of the cell types in the CoNSeP images (Fig. 5d). In both examples, the first principal component highlighted malignant epithelium (red) cells. The second principal component, respectively, highlighted miscellaneous cells (yellow) and inflammatory (magenta) cells. DINO v.2 was shown to learn a similar semantic feature separation on natural images, allowing foreground/background separation (for example, discriminating a bus or a bird from the background) as well as part annotation (for example, wheels versus windows in a bus)^33^. Here, we show that this emerging property of the model carries over to the pathology domain. This encouraging result supports our expectation that the unsupervised features learned by Virchow are meaningful and interpretable for a wide range of downstream tasks.

## Discussion

The value of a pathology foundation model is twofold: generalizability and training data efficiency. In our study, we demonstrate both of these benefits. Virchow-based pan-cancer prediction generalized well to tissue types or slides submitted from institutions not observed in the training data. Rare histological subtypes of cancer were detected nearly as well as common variants. The same pan-cancer detection model was shown to almost match the performance of clinical-grade models overall (AUC from 0.001 to 0.007 behind clinical products, *P* < 0.01) and surpassed them in the detection of some rare variants of cancers, despite training with fewer tissue-specific labels. This result is even more impressive when noting that the training dataset of the pan-cancer model, as a proof of concept, lacks the quality control and subpopulation enrichment of data and labels that are typically done for commercially available AI models. Finally, we note that Virchow embeddings were not fine-tuned, and models used simple aggregator architectures to make predictions. These results build confidence that, with sufficient scale, foundation models will serve as the building blocks for the future development of a wide variety of downstream tasks.

There are a few areas in which we anticipate particularly high-value impact. In clinical practice, where most biopsy samples are benign, a pan-cancer detection system can prioritize cases to help reduce diagnostic turnaround. With decreasing training data requirements, clinical-grade products for less common cancers could be developed. Biomarker prediction using routine H&E WSIs would increase screening rates; reduce intrusive, tissue-destructive testing; and rapidly provide the data needed to make more informed treatment decisions. Virchow embeddings demonstrated sufficiently high performance to suggest these tools are achievable. Indeed, Virchow unlocks the ability to accurately and precisely detect unusual histological variants of cancer as well as biomarker status, something that is difficult to achieve with cancer- or biomarker-specific training due to the limited amount of associated training data.

Despite the observed improvements, there are still aspects of Virchow’s development that merit further discussion. Histopathology data differs from natural image data in key ways: the long-tailed distribution of pathologic entities and histological structures, the lack of object scale diversity and the restricted color space. Self-supervised learning algorithms attempt to match the inductive biases of the learning algorithm to the data distribution; however, in this work, as in many other works in self-supervised learning for computational pathology, algorithmic and training settings are largely based on what was successful in the natural image domain. Further study may reveal that altering these design choices will further improve performance in the pathology domain.

It remains an open question at what point the model and data scale are saturated. We found that pan-cancer detection performance scales with model and dataset size (Fig. 2g), which is consistent with observations of prior foundation models in other domains^28–30^. The improvement in performance with respect to model size appears to still be in an approximately log-linear range; however, sub-log-linear trends were observed as a function of training data. Trends in training data size may be oversimplified as they do not capture the tradeoff between increasing the number of WSIs versus tiles. The setting is too complex to draw precise conclusions about the effect of dataset diversity, although we posit that increased diversity helps to learn robust and rare features. Indeed, it has been shown that training a model on multiple tissues or cancer variants can improve detection performance for each cancer^53^, as many morphological features are observed across cancers from different topographies^54^. Overall, our investigation into scaling behavior suggests that increasing the number of model parameters remains a salient axis to explore.

Our work has several limitations. The training dataset is acquired from one center with limited scanner types. As with most histopathology self-supervised models, embeddings are generated at the tile level using ×20 magnification (0.5 mpp) as opposed to the slide level and therefore require training an aggregation model. Although scaling up the size of a tile-level foundation model may improve performance, it is likely that such models must be extended to the slide level to achieve the data efficiency required for low-data tasks such as the prediction of biomarkers, treatment response or clinical outcome. A deep investigation of aggregator architectures and training procedures is beyond the scope of this work. As is the case for all models aiming for clinical application, thorough stratified performance validation is required. Furthermore, hardware considerations must be made toward the deployment of models the size of Virchow or larger; model distillation may be appropriate for some tasks. Due to the scale of training, our study has not been able to fully explore the effectiveness of data-balancing and -distillation strategies. The challenge of curating training data that preserves rare features while reducing redundancy remains an open question. Considering the long-tail distribution in digital pathology, we question the suitability of clustering-based data distillation methods such as those used in the original DINO v.2 model for natural images^33^.

Recent advances in computational pathology have been supported by increased dataset scale and reduced reliance on labels. Using multiple-instance learning^14,49,55^ with labels at the level of groups of slides has enabled clinically relevant diagnostics by scaling to training datasets on the order of 10,000 WSIs^10–14^. These earlier works typically initialized the model’s embedding parameters using pretrained model weights, often those trained on ImageNet in a supervised setting. This process, called transfer learning, was motivated by the observation that model performance critically depends on the model’s ability to capture image features. In-domain transfer learning was not possible given the limited availability of labeled pathology datasets. Now self-supervised learning is enabling in-domain transfer by removing the label requirement, driving a second wave of scaling to tens of thousands of WSIs to inform image representation^35–39,56^. Virchow marks a major increase in training data scale to 1.5 million WSIs—a volume of data that is over 3,000 times the size of ImageNet^31^ as measured by the total number of pixels. This large scale of data in turn motivates large models that can capture the diversity of image features in WSIs. In this work, we have demonstrated that this approach can form the foundation for clinical-grade models in cancer pathology.

## Methods

### Million-scale training dataset

Institutional review board review was not applicable for the research described in this study. This research study was conducted retrospectively from deidentified data licensed to Paige.AI, Inc. from MSKCC. The data used in this study were all collected originally for clinical use by MSKCC in the practice setting and are therefore considered secondary data. Only data previously deidentified by MSKCC were utilized in the analysis, and unique patient identifiers were completely removed from the analytical dataset. To the best of our knowledge, MSKCC has not transferred any data for which the applicable patient has not consented to or otherwise agreed to MSKCC’s Notice of Privacy Practices or a substantially similar notice, waiver or consent. The training digital pathology dataset comprises 1,488,550 WSIs derived from 119,629 patients. These WSIs are all stained with H&E, a routine stain that stains the nuclei blue and the extracellular matrix and cytoplasm pink. The WSIs are scanned at ×20 resolution or 0.5 mpp using Leica scanners. Seventeen high-level tissue groups are included, as illustrated in Fig. 1c.

WSIs are gigapixels in size and are challenging to use directly during training. Instead, Virchow was trained on tissue tiles that were sampled from foreground tissue in each WSI. To detect foreground, each WSI was downsampled 16× with bilinear interpolation, and every pixel of the downsampled image was evaluated as to whether its hue, saturation and value were within [90, 180], [8, 255] and [103, 255], respectively. All non-overlapping 224 × 244 tiles containing at least 25% tissue by area were collected. Virchow was trained on 2 billion tiles sampled randomly with replacement from approximately 13 billion available tissue tiles.

### Virchow architecture and training

**Extended Data Fig. 1:**
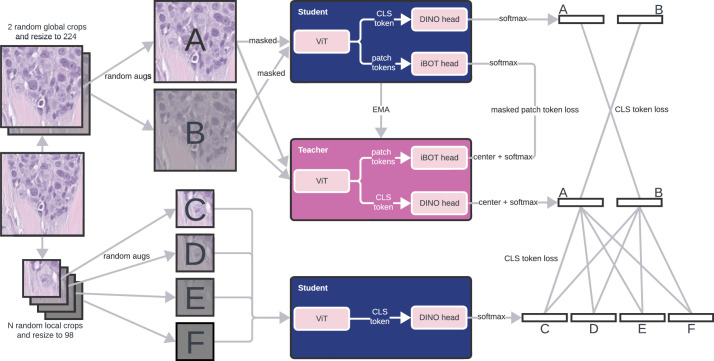
Schematic of the Agata aggregator. The Agata aggregator learns to attend to tiles that contribute toward the label decision using cross-attention. The operation is defined using query Q, key K, and value matrix V: ${{{\rm{softmax}}}}(\frac{Q{K}^{T}}{\sqrt{{d}_{k}}})V$, where *d*_*k*_ is the output dimension of the key matrix. In contrast to the typical self-attention mechanism where Q, K, V are projected from the inputs, Q is parameterized directly by the model to reduce GPU memory consumption. When aggregating across the tens or hundreds of thousands of tiles in a specimen, full attention requires too much GPU memory. This simplified attention can be interpreted as a learned weighted sum of all tile-level features. Indeed, full self-attention is quadratic in memory with respect to the number of tiles whereas the attention in Agata is linear. K and V are obtained with two consecutive Gaussian Error Linear Unit (GELU) projection layers as: $K=GELU({W}_{1}^{T}x+{b}_{1})$ and $V=GELU({W}_{2}^{T}K+{b}_{2})$, where x is the tile embedding, and *W*_*n*_, *b*_*n*_ are the weight and bias parameters for the projection layers. In our experiments, *W*_1_ produces 256-dimensional keys, *W*_2_ produces 512-dimensional values, and we omit scaling by $\sqrt{\{{d}_{k}\}}=16$. After the attention step, two linear layers with non-linear activation (ReLU) are used followed by a final linear layer with softmax activation.

Virchow employs the ViT ‘huge’ architecture (ViT-H/14), a ViT^34^ with 632 million parameters that was trained using the DINO v.2 (ref. ^33^) self-supervised learning algorithm, as illustrated in Extended Data Fig. 1. The ViT is an adaptation of the transformer model for image analysis, treating an image as a sequence of patches. These patches are embedded and processed through a transformer encoder that uses self-attention mechanisms. This approach allows ViT to capture complex spatial relationships across the image. DINO v.2 is based on a student–teacher paradigm: given a student network and a teacher network, each using the same architecture, the student is trained to match the representation of the teacher. The student network is information-limited, as it is trained using noisy variations of input tiles. The teacher network is a slowly updated exponential moving average of past student networks; matching the teacher achieves an effect similar to ensembling over prior student predictions^57^. The student learns a global representation of an image by matching the teacher’s class token, as well as local representations by matching the teacher’s patch tokens. Patch tokens are only matched for a select subset of tokens that are randomly masked out of an input image (for the student), as done in masked image modeling^58^. Additional regularization helps DINO v.2 trained models outperform the earlier DINO variant^25^.

The default hyperparameters for training the DINO v.2 model were used for Virchow as detailed in ref. ^33^ with the following changes: a teacher temperature schedule of 0.04–0.07 in 186,000 iterations and a reciprocal square root learning rate schedule with a warmup of 495,000 iterations (instead of 100,000) and linear cooldown to 0.0 for the last 819,200 iterations^29^. Virchow was trained using AdamW (*β*_1_ = 0.9, *β*_2_ = 0.999) with float16 precision. Note that with ViT-H, we used 131,072 prototypes (and thus 131,072-dimensional projection heads). During distributed training, each mini-batch was sampled by randomly selecting one WSI per graphics processing unit and 256 foreground tiles per WSI.

### Pan-cancer detection

**Extended Data Fig. 2:**
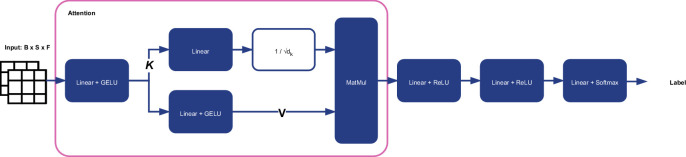
Schematic of the DINOv2 training routine. Virchow used a ViT-H architecture, trained with DINOv2. From a single tile, 2 global crops and 8 local crops all with random augmentations are created. The global crops are randomly masked and fed to the student model, and the unmasked versions are fed to the teacher model. The student tries to produce a global representation of the views (via the CLS token) that matches the teacher’s representation of the opposite view. The student also tries to produce representations of the masked image tokens that match the teacher’s representations of the same tokens but unmasked. The local crops are only fed to the student which tries to produce a representation that matches the teacher’s representations of the global crops. The teacher is an exponential moving average (EMA) copy of the student.

Specimen-level pan-cancer detection requires a model that aggregates foundation model embeddings from all foreground tiles of all WSIs in a specimen to detect the presence of cancer. All pan-cancer detection models trained in this work use an Agata^10^ aggregator model, weakly supervised with multiple-instance learning (see Extended Data Fig. 2 for architecture details).

#### Embedding generation

For a 224 × 224 input tile image, a Virchow embedding is defined as the concatenation of the class token and the mean across all 256 of the other predicted tokens. This produces an embedding size of 2,560 (1,280 × 2). For Phikon, only the class token is used, as recommended by ref. ^37^. For CTransPath, the mean of all tokens is used as there is no class token.

#### Training data

To train the aggregator model, we prepared a subset of the training dataset used for training Virchow (see ‘Million-scale training dataset’ in Methods for details), combined with specimen-level labels (block-level for prostate tissue) indicating the presence or absence of cancer extracted from synoptic and diagnostic reports. The training and validation datasets combined consist of 89,417 slides across 40,402 specimens. See Extended Data Fig. 4b for the training data distribution, stratified by WSI tissue type and cancer status.

#### Aggregator training

The Agata aggregator was trained as described in Extended Data Fig. 2. Because the label is at the level of the specimen, all tiles belonging to the same specimen need to be aggregated during training. Training using embeddings for all tiles of a specimen is prohibitively memory-intensive. We thus select the slide with the highest predicted cancer probability per specimen and backpropagate the gradients only for that slide.

As baselines, aggregators using Phikon and CTransPath embeddings were also trained. All aggregators were trained for 25 epochs using the cross-entropy loss and the AdamW^59^ optimizer with a base learning rate of 0.0003. During each training run, the checkpoint with the highest validation AUC was selected for evaluation.

#### Testing dataset

The pan-cancer detection models are evaluated on a combination of data sourced from MSKCC and external institutions. None of the patients in the evaluation set were seen during training. The dataset contains 22,932 slides from 6,142 specimens across 16 cancer types. We hypothesize that the more data the foundation model is trained on, the better the downstream task performance, especially on data-constrained tasks. To test this hypothesis, we categorize cancer types into common or rare cancer groups. According to the NCI, rare cancers are defined as those occurring in fewer than 15 people out of 100,000 each year in the United States^46^. Based on this definition, common cancer comprises 14,179 slides from 3,547 specimens originating in breast, prostate, lung, colon, skin, bladder, uterus, pancreas and H&N, and rare cancer comprises 8,753 slides from 2,595 specimens originating in liver, stomach, brain, ovary, cervix, testis and bone. Note that each cancer type is determined by its tissue of origin and thus may appear in any tissue (as primary or metastatic cancer). On the other hand, benign specimens for each cancer type were sampled only from the tissue of origin. For example, the liver stratum contains 182 liver specimens with liver cancer (primary), 18 non-liver specimens with liver cancer (metastatic) and 200 benign liver specimens. For each cancer type, Fig. 2a shows the distribution between primary and metastatic cancer, and Extended Data Fig. 4a additionally shows the number of benign specimens.

The testing dataset includes 15,622 slides from 3,033 specimens collected at MSKCC (denoted as ‘Internal’ in Fig. 2b), in addition to 7,310 slides (3109 specimens) sent to MSKCC from institutions around the world (‘External’ in Fig. 2b). See Extended Data Fig. 4a for the testing data distribution, stratified by cancer type (for specimens with cancer) or by tissue type (for benign specimens).

#### Label extraction

To establish the clinical cancer diagnosis at the specimen level, a rule-based natural language processing system was employed. This system decomposes case-level reports to the specimen level and analyzes the associated clinical reports with each specimen, thereby providing a comprehensive understanding of each case.

#### Statistical analysis

**Extended Data Fig. 3:**
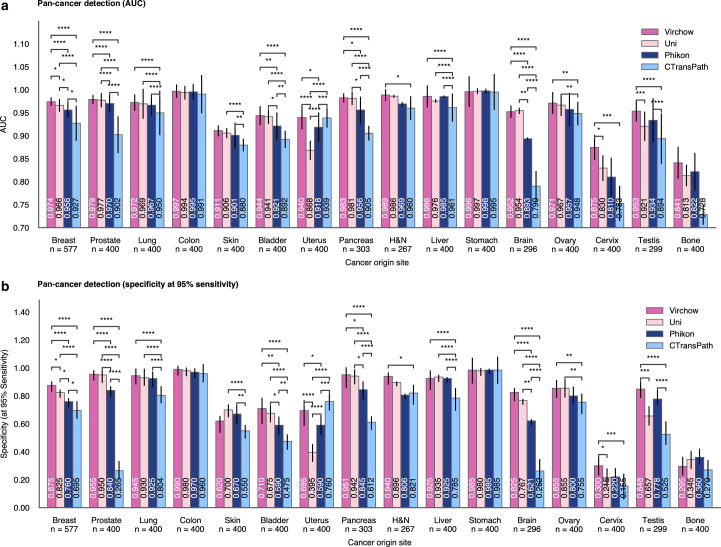
Pan-cancer detection results for every cancer origin site. **a**. Area under (the receiver operator characteristic) curve (AUC); **b**. specificity at 95% sensitivity. H&N is head and neck. In both plots, a pairwise comparison of statistical significance is computed using the pairwise DeLong’s test for AUC and Cochran’s Q test followed by McNemar’s test for specificity, both corrected for multiple comparisons with Holm’s method (* p < 0.05, ** p < 0.01, *** p < 0.001, **** p < 0.0001). Error bars show the two-sided 95% confidence interval computed with DeLong’s method for AUC and Wilson’s method for specificity.

**Extended Data Fig. 4:**
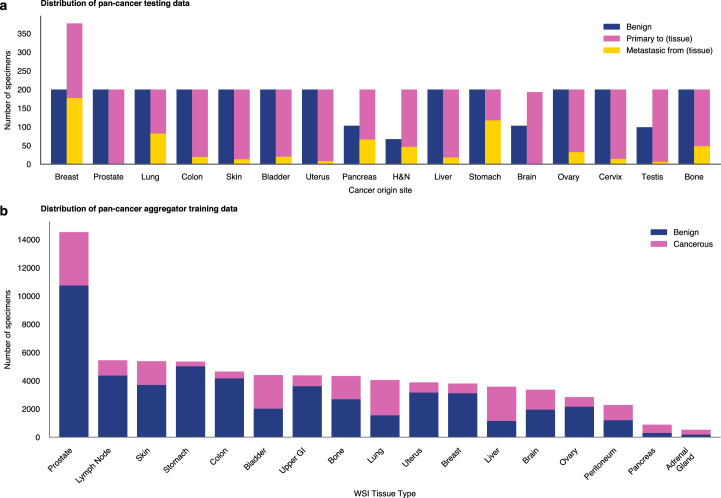
Pan-cancer dataset distribution. **a**. Specimen counts per cancer origin site in the pan-cancer testing dataset (H&N is head and neck). **b**. Specimen counts per tissue type in the pan-cancer aggregator training dataset.

The performance of the three models is compared using two metrics: AUC and specificity at 95% sensitivity. AUC is a suitable general metric because it does not require selecting a threshold for the model’s probability outputs, something that may need tuning for different data subpopulations. Specificity at 95% sensitivity is informative because a clinical system must be not only sensitive but also specific in practice. For AUC, the pairwise DeLong’s test^60^ with Holm’s method^61^ for correction is applied to check for statistical significance. For specificity, first Cochran’s *Q* test^62^ is applied, and then McNemar’s test^63^ is applied post hoc for all pairs with Holm’s method for correction. The two-sided 95% confidence intervals in Fig. 2b–e and Extended Data Fig. 3 were calculated using DeLong’s method^60^ for AUC and Wilson’s method^64^ for specificity. In addition to overall analysis, stratified analysis is also conducted for each cancer type.

### Clinical evaluation datasets

To perform an extensive evaluation of the Virchow-based pan-cancer detection model, we employ seven additional datasets (see Supplementary Table 2.1 for details). One of these datasets is pan-tissue, and the rest are single-tissue datasets containing tissues for which Paige has clinical products: that is, prostate, breast and lymph node.

#### Pan-tissue product benchmark

This dataset contains 2,419 slides across 18 tissue types (Supplementary Table 2.2). Each slide is individually inspected by a pathologist and labeled according to presence of invasive cancer. An important distinction between the testing dataset in ‘Pan-cancer detection’ and this dataset is that the former is stratified according to origin tissue in cancerous specimens, whereas the latter is stratified according to tissue type for all slides, as it is more relevant in a clinical setting. We use this dataset to identify failure modes of the pan-cancer detection model.

#### Prostate product benchmark

This dataset contains 2,947 blocks (3,327 slides) of prostate needle core biopsies (Supplementary Table 2.7). Labels for the blocks are extracted from synoptic reports collected at MSKCC. This dataset has been curated to evaluate the standalone performance of Paige Prostate Detect, which is a tissue-specific, clinical-grade model. We use this dataset to compare the pan-cancer detection model to Paige Prostate Detect.

#### Prostate rare variants benchmark

This dataset contains 28 slides containing rare variants of prostate cancer (neuroendocrine tumor, atrophic, small lymphocytic lymphoma, foamy cell carcinoma, follicular lymphoma) and 112 benign slides (Supplementary Table 2.8). Cancerous slides are curated and labeled by a pathologist, and are appended with slides from benign blocks determined from synoptic reports collected at MSKCC.

#### Breast product benchmark

This dataset contains 190 slides with invasive cancer and 1,501 benign slides, labeled individually by a pathologist according to presence of atypical ductal hyperplasia, atypical lobular hyperplasia, lobular carcinoma in situ, ductal carcinoma in situ, invasive ductal carcinoma, invasive lobular carcinoma and/or other subtypes (Supplementary Table 2.5). This dataset has been curated to evaluate the standalone performance of Paige Breast, which is a tissue-specific, clinical-grade model. We use the subtype information for stratified analysis.

#### Breast rare variants benchmark

This dataset contains 23 cases of invasive ductal carcinoma or invasive lobular carcinoma (as control), 75 cases of rare variants (adenoid cystic carcinoma, carcinoma with apocrine differentiation, cribriform carcinoma, invasive micropapillary carcinoma, metaplastic carcinoma (matrix producting subtype, spindle cell and squamous cell), mucinous carcinoma, secretory carcinoma and tubular carcinoma) and 392 benign cases (total 5,031 slides). Cancerous cases are curated by a pathologist, and are appended with benign cases determined from synoptic reports collected at MSKCC. See Supplementary Table 2.6 for details.

#### BLN

This dataset contains 458 lymph node slides with metastasized breast cancer and 295 benign lymph node slides (Supplementary Table 2.3). Each slide has been labeled by a pathologist according to presence of invasive cancer, and the largest tumor on the slide is measured to categorize the tumor into macrometastasis, micrometastasis or infiltrating tumor cells. We use the categories for stratified evaluation.

#### Lymph node rare variants benchmark

This dataset contains 48 specimens of rare variants of cancers (diffused large B-cell lymphoma, follicular lymphoma, marginal zone lymphoma, Hodgkin’s lymphoma) selected by a pathologist and 192 benign specimens determined from synoptic reports collected at MSKCC (Supplementary Table 2.4).

### Biomarker detection

We formulated each biomarker prediction task as a binary pathology case classification problem, where a positive label indicates the presence of the biomarker. Each case consists of one or more H&E slides that share the same binary label. We randomly split each dataset into training and testing subsets, ensuring no patient overlap, as shown in Supplementary Table 3.1. The clinical importance of each biomarker is described below.

#### Colon-MSI

Microsatellite instability (MSI) occurs when DNA regions with short, repeated sequences (microsatellites) are disrupted by single nucleotide mutations, leading to variation in these sequences across cells. Normally, mismatch repair (MMR) genes (*MSH1*, *MSH2*, *MSH6*, *PMS2*) correct these mutations, maintaining consistency in microsatellites. However, inactivation of any MMR gene (through germline mutation, somatic mutation or epigenetic silencing) results in an increased rate of uncorrected mutations across the genome. MSI is detected using polymerase chain reaction or next-generation sequencing, which identifies a high number of unrepaired mutations in microsatellites, indicative of deficient mismatch repair (dMMR). Microsatellite instability high (MSI-H) suggests dMMR in cells, identifiable via IHC, which shows absent staining for MMR proteins. MSI-H is present in approximately 15% of colorectal cancers (CRCs), often linked to germline mutations that elevate hereditary cancer risk. Consequently, routine MSI or IHC-based dMMR screening is recommended for all primary colorectal carcinoma samples. The Colon-MSI dataset, comprising 2,698 CRC samples with 288 MSI-H/dMMR positive cases, uses both IHC and MSK-IMPACT sequencing for dMMR and MSI-H detection, prioritizing IHC results when both test outcomes are available.

#### Breast-CDH1

The biallelic loss of cadherin 1 (*CDH1*) gene (encoding E-cadherin) is strongly correlated with lobular breast cancer and a distinct histologic phenotype and biologic behavior^65^. *CDH1* inactivating mutations associated with loss of heterozygosity or a second somatic loss-of-function mutation as determined by MSK-IMPACT sequencing test results were considered as ‘*CDH1* biallelic mutations’. The CDH1 dataset comprises a total of 1,077 estrogen receptor-positive (ER+) primary breast cancer samples, in which 139 were positive and 918 were negative. The remaining 20 samples with other types of variants—that is, monoallelic mutations—were excluded.

#### Bladder-FGFR

The fibroblast growth factor receptor (FGFR) is encoded by four genes (*FGFR1*, *FGFR2*, *FGFR3*, *FGFR4*). FGFR gene alterations screening in bladder carcinoma allows the identification of patients targetable by FGFR inhibitors. Anecdotal experience from pathologists suggested there may be a morphological signal for FGFR alterations^66^. The FGFR binary label focuses on *FGFR3 p.S249C*, *p.R248C*, *p.Y373C*, *p.G370C* mutations, *FGFR3*-*TACC3* fusions and *FGFR2 p.N549H*, *pN549K*, *p.N549S*, *p.N549T* mutations based on data from the MSK-IMPACT cohort. From the total of 1,038 samples (1,087 WSIs), 26.2% have *FGFR3* alterations.

#### Lung-EGFR

The *EGFR* oncogenic mutation screening in non-small cell lung cancer is essential to determine eligibility for targeted therapies in late stage non-small cell lung cancer^67^. The oncogenic status of *EGFR* mutation was determined based on OncoKB annotation^68^. *EGFR* mutations with any oncogenic effect (including predicted/likely oncogenic) were defined as positive label, and *EGFR* mutation with unknown oncogenic status were excluded.

#### Prostate-AR

The *AR* amplification/overexpression was found in 30%–50% of castration resistant prostate cancers and was associated with resistance to androgen deprivation therapy. In the AR dataset, the copy number amplification of *AR* was determined by MSK-IMPACT sequencing test, for which the fold change was greater than two.

#### Gastric-HER2

Human epidermal growth factor receptor 2 (*HER2*) overexpression and/or amplification are much more heterogeneous in gastric cancer compared to breast cancer. Approximate 20% of gastric cancer patients are found to correlate with *HER2* overexpression/high-level amplification, and they would be likely to benefit from treatment with an anti-HER2 antibody therapy. Here, a *HER2* IHC result of 2+, confirmed positive with fluorescence in situ hybridization (FISH) or an IHC result of 3+ were considered *HER2* amplification.

#### Endometrial-PTEN

*PTEN* is the most frequently mutated tumor suppressor gene in endometrial cancer. The presence of *PTEN* mutation showed to be significantly associated with poorer prognosis in survival and disease recurrence. The oncogenic status of *PTEN* mutation was determined based on MSK-IMPACT sequencing and OncoKB annotation^68^. The variants associated with any oncogenic effect (including predicted and/or likely oncogenic) were defined as positive label for *PTEN* mutations, and variants with unknown oncogenic status were excluded.

#### Thyroid-RET

*RET* mutations were highly associated with medullary thyroid cancer, which accounts for about 5–10% of all thyroid cancer. Screening *RET* oncogenic mutations plays an important role in diagnosis and prognosis of medullary thyroid cancer. The positive label for *RET* oncogenic mutation was determined by MSK-IMPACT sequencing and OncoKB annotation^68^.

#### Skin-BRAF

*BRAF* is one of the most frequently mutated genes in melanoma, and V600E mutation is the most common variant, which leads to constitutive activation of the BRAF/MEK/ERK signaling pathway. Targeted therapy with BRAF inhibitors showed better survival outcome in patients with *BRAF* V600-mutated melanoma. Therefore, the detection of *BRAF* V600 mutations in melanoma helps to determine treatment strategies. In the BRAF dataset, the oncogenic mutation status and the presence of V600E variant were determined based on the MSK-IMPACT cohort and OncoKB annotation^68^.

#### Ovarian-FGA

High-grade serous ovarian cancer is characterized by high prevalence of *TP53* mutations and genome instability with widespread genetic alteration. The fraction of genome altered (FGA) was determined from MSK-IMPACT sequencing data, where FGA ≥ 30% was treated as a positive label. A cut-off for FGA was established that enriched for *TP53* mutations in the distribution of ovarian cancer cases.

#### Aggregator training

For weakly supervised biomarker prediction, we used embeddings and Agata^10^, as in ‘Pan-cancer detection’, to transform a set of tiles extracted from WSIs that belong to the same case to the case-level target label. Virchow is used to generate tile-level embeddings on all the evaluated datasets with 224 × 224 resolution at ×20 magnification. To thoroughly compare the quality of the embeddings, we trained an aggregator for learning rates in 1 × 10^−4^, 5 × 10^−5^, 1 × 10 ^−5^, 5 × 10^−6^, 1 × 10^−6^ and report the best observed test AUC scores in Fig. 4b. Due to the small biomarker dataset sizes, the learning rate was not chosen on a validation set to evaluate generalization; rather, this serves as a benchmark across the different types of tile embeddings (Virchow, UNI, Phikon and CTransPath), yielding an estimate of the best possible biomarker performance for each type.

#### Statistical analysis

AUC is used to compare models without having to select a threshold on the models’ predicted probability values, which may differ by data subpopulation. The two-sided 95% confidence intervals in Fig. 4b are calculated using DeLong’s method^60^.

### Tile-level benchmarking

For evaluating Virchow on tile-sized images, the linear probing protocol, as well as dataset descriptions and the statistical analysis, are described below. Dataset details, including training, validation, and testing splits, are also summarized in Supplementary Table 4.1.

#### Linear probing protocol

For each experiment, we trained a linear tile classifier with a batch size of 4,096 using the stochastic gradient descent optimizer with a cosine learning rate schedule, from 0.01 to 0, for 12,500 iterations, on top of embeddings generated by a frozen encoder. The large number of iterations is intended to allow any linear classifier to converge as far as it can at each learning rate step along the learning rate schedule. All embeddings were normalized by *Z*-scoring before classification. Linear probing experiments did not use data augmentation. For testing set evaluation, the classifier checkpoint that achieved the lowest loss on the validation set was selected. A validation set was used for all tasks. If one was not provided with the public dataset, we randomly split out 10% of the training data to make a validation set.

#### PanMSK

For a comprehensive in-distribution benchmark, 3,999 slides across the 17 tissue types in Fig. 1d were held out from the training dataset collected from MSKCC. Of these, 1,456 contained cancer that was either partially or exhaustively annotated with segmentation masks by pathologists. These annotations were used to create a tile-level dataset of cancer versus non-cancer classification, which we refer to as PanMSK. All images in PanMSK are 224 × 224 pixel tiles at 0.5 mpp. See Supplementary Note 5 for further details.

#### CRC

The CRC classification public dataset^69^ contains 100,000 images for training (from which we randomly selected 10,000 for validation) and 7,180 images for testing (224 × 224 pixels) at ×20 magnification sorted into nine morphological classes. Analysis is performed with both the Macenko-stain-normalized (NCT-CRC-HE-100K) and unnormalized (NCT-CRC-HE-100K-NONORM) variants of the dataset. It should be noted that the training set is normalized in both cases, and only the testing test is unnormalized in the latter variant. Thus, the unnormalized variant of CRC involves a distribution shift from training to testing.

#### WILDS

The Camelyon17-WILDS dataset is a public dataset comprising 455,954 images, each with a resolution of 96 × 96 pixels, taken at ×10 magnification and downsampled from ×40. This dataset is derived from the larger Camelyon17 dataset and focuses on lymph node metastases. Each image in the dataset is annotated with a binary label indicating the presence or absence of a tumor within the central 32 × 32 pixel region. Uniquely designed to test OOD generalization, the training set (335,996 images) is composed of data from three different hospitals, whereas the validation subset (34,904 images) and testing subset (85,054 images) each originate from separate hospitals not represented in the training data.

#### MHIST

The colorectal polyp classification public dataset (MHIST^70^) contains 3,152 images (224 × 224 pixels) presenting either hyperplastic polyp or sessile serrated adenoma at ×5 magnification (downsampled from ×40 to increase the field of view). This dataset contains 2,175 images in the training subset (of which we randomly selected 217 for validation) and 977 images in the testing subset.

#### TCGA TIL

The TCGA TIL public dataset is composed of 304,097 images (100 × 100 pixels) at ×20 magnification^71–73^, split into 247,822 training images, 38,601 validation images and 56,275 testing images. Images are considered positive for tumor-infiltrating lymphocytes if at least two TILs are present and labeled negative otherwise. We upsampled the images to 224 × 224 to use with Virchow.

#### PCam

The PatchCamelyon (PCam) public dataset consists of 327,680 images (96 × 96 pixels) at ×10 magnification, downsampled from ×40 to increase the field of view^9,74^. The data is split into a training subset (262,144 images), a validation subset (32,768 images), and a testing subset (32,768 images). Images are labeled as either cancer or benign. We upsampled the images to 224 × 224 pixels to use with Virchow.

#### MIDOG

The MIDOG public dataset consists of 21,806 mitotic and non-mitotic events labeled on 503 7,000 × 5,000 WSI regions from several tumor, species and scanner types^75^. Data was converted into a binary classification task by expanding each 50 × 50 pixel annotation to 224 × 224 regions and then randomly shifting in the horizontal and vertical regions such that the event is not centered in the tile. All negative instances that overlapped with positive instances were removed from the dataset. The resulting dataset consists of training, validation and testing subsets with 13,107, 4,359 and 4,340 images, respectively (of which 6,720, 2,249 and 2,222 have mitotic events, respectively, and the rest contain confounders that mimic mitotic events).

#### TCGA CRC-MSI

The TCGA CRC-MSI classification public dataset consists of 51,918 512 × 512 regions taken at ×20 magnification presenting colorectal adenocarcinoma samples^76^. Samples were extracted and annotated from TCGA. Regions were labeled either as microsatellite-instable or microsatellite-stable. We downsampled regions to 448 × 448 to use with Virchow.

#### Statistical analysis

The (weighted) *F*1 score is used to compare models as this metric is robust to class imbalance. Accuracy and balanced accuracy are also computed, as described in Supplementary Note 4. The two-sided 95% confidence intervals in Fig. 5 and Supplementary Table 4.2 were computed with 1,000 bootstrapping iterations over the metrics on the testing set without retraining the classifier. McNemar’s test was used to determine statistically significant (*P* < 0.05) differences between results.

### Qualitative feature analysis

We performed an unsupervised feature analysis similar to the procedure in ref. ^33^, using the CoNSeP dataset^52^ of H&E stained slides with colorectal adenocarcinoma. CoNSeP provides nuclear annotations of cells in the following seven categories: normal epithelial, malignant/dysplastic epithelial, fibroblast, muscle, inflammatory, endothelial and miscellaneous (including necrotic, mitotic and cells that couldn’t be categorized). Because CoNSeP images are of size 1,000 × 1,000 and Virchow takes in images of size 224 × 224, we resized images to 896 × 896 and divided them into a 4 × 4 grid of non-overlapping 224 × 224 subimages before extracting tile-level features. For a given image, we used principal component analysis (PCA) on all the tile features from the subimages, normalized the first and second principal components to values within [0, 1] and thresholded at 0.5. Figure 5d shows some examples of the unsupervised feature separation achieved in this way.

### Software

For data collection, we used Python (v.3.10.11) along with Pandas (v.2.2.2) for indexing the data and metadata used for pretraining and benchmarking. OpenSlide (v.1.3.1) and Pillow (v.10.0.0) were used for preprocessing the image tiles for the benchmark. Where appropriate, we extracted per-specimen labels from clinical reports using DBT (v.1.5.0). We used Python (v.3.10.11) for all experiments and analyses in the study, which can be replicated using open-source libraries as outlined below. For self-supervised pretraining, we used PyTorch (v.2.0.1) and Torchvision (v.0.15.1). The DINO v.2 code was ported from the official repository (https://github.com/facebookresearch/dinov2) and adapted to PyTorch Lightning (v.1.9.0). All WSI processing during pretraining was performed online and was supported by cucim (v.23.10.0) and torchvision (v.0.16.1). For downstream task benchmarking, we use scikit-learn (v.1.4.2) for logistic regression and metrics computation. Implementations of other pretrained visual encoders benchmarked in the study were obtained from the following links: UNI (https://huggingface.co/MahmoodLab/UNI), Phikon (https://huggingface.co/owkin/phikon), DINOp=8 (https://github.com/lunit-io/benchmark-ssl-pathology), PLIP (https://huggingface.co/vinid/plip), CTransPath (https://github.com/Xiyue-Wang/TransPath) and the original natural image pretrained DINO v.2 (https://github.com/facebookresearch/dinov2).

### Reporting summary

Further information on research design is available in the Nature Portfolio Reporting Summary linked to this article.

## Online content

Any methods, additional references, Nature Portfolio reporting summaries, source data, extended data, supplementary information, acknowledgements, peer review information; details of author contributions and competing interests; and statements of data and code availability are available at 10.1038/s41591-024-03141-0.

## Supplementary information

Supplementary Information The document is split into ‘Supplementary Notes’ sections that may or may not contain descriptive text along with tables and figures. Section 1 ‘Early foundation models in computational pathology’ describes pathology foundation models in the literature. Table 1.1 summarizes these models. Section 2 ‘Clinical Evaluation’, Table 2.1 summarizes datasets used in clinical eval of Virchow (results in Fig. 3). Tables 2.2–2.8: per-dataset tables detailing stratified sample counts for the datasets in Table 2.1. Section 3: Table 3.1 describes slide- and case-level sample counts for the biomarker prediction datasets (train/tune/test). Section 4 ‘Tile-level benchmarks’ describes additional metrics used to evaluate tile-level linear probing results. Table 4.1 describes the tile benchmark datasets. Table 4.2: tile-level results (additional metrics). Table 4.3: TCGA CRC-MSI tile-level biomarker prediction results (this task is not in the main figure). Table 4.4: PCAM and WILDS using a pretrained CNN from a breast-specialized model. Section 5 ‘Multitissue PanMSK dataset’ describes the preparation of the internal PanMSK tile-level benchmark dataset in detail. Table 5.1: PanMSK data splits. Fig. 5.1: tile distribution (tissues, cancer/benign).

Reporting Summary

## Data Availability

This study did not specifically collect patient data. The retrospective analysis utilized proprietary deidentified digital pathology whole slides and associated metadata were exclusively licensed by Paige.AI, Inc. from MSKCC. Requests for data need to be submitted to Paige AI (https://paige.ai/contact-us/) and evaluated by Paige AI and MSKCC on a case-by-case basis. All requests complying with internal regulations on data privacy and intellectual property will be granted. This study also utilized the following publicly available datasets for downstream benchmarking: CRC (NCT-CRC-HE-100K and NCT-CRC-HE-100K-NONORM, available via Zenodo at https://zenodo.org/records/1214456 (ref. ^77^)), WILDS (Camelyon17; https://wilds.stanford.edu/get_started), PCam (https://github.com/basveeling/pcam), MHIST (https://bmirds.github.io/MHIST), TCGA TIL (available via Zenodo at https://zenodo.org/records/6604094 (ref. ^71^)), MIDOG (https://midog.deepmicroscopy.org/download-dataset/) and TCGA CRC-MSI (available via Zenodo at https://zenodo.org/records/3832231 (ref. ^76^)).

## Code availability

The model can be accessed for non-commercial research and replication of the results reported in this manuscript at https://huggingface.co/paige-ai/Virchow. To help researchers use our model, a public software development kit for leveraging foundation model embeddings to develop downstream WSI applications is available at https://github.com/Paige-AI/paige-ml-sdk. We have documented all experiments with sufficient details in our Methods section to enable independent replication. Although the full codebase cannot be shared due to dependencies on proprietary libraries and specific hardware configurations, such as distributed clusters and storage, the core components of our work rely on open-source repositories. These include the DINO v.2 model architecture used for self-supervised pretraining (https://github.com/facebookresearch/dinov2), as well as the PyTorch Lightning training framework (https://github.com/Lightning-AI/pytorch-lightning) and the underlying torch library (https://github.com/pytorch/pytorch) used for training and inference with this model.
