# Supplementary material for: A foundation model for clinical-grade computational pathology and rare cancers detection
Source: Nat Med. 2024 Jul 22;30(10):2924–35. doi: 10.1038/s41591-024-03141-0 (PMC11485232; doi:10.1038/s41591-024-03141-0)
Supplement: Supplementary file 1 — The document is split into ‘Supplementary Notes’ sections that may or may not contain descriptive text along with tables and figures. Section 1 ‘Early foundation models in computational pathology’ describes pathology foundation models in the literature. Table 1.1 summarizes these models. Section 2 ‘Clinical Evaluation’, Table 2.1 summarizes datasets used in clinical eval of Virchow (results in Fig. 3). Tables 2.2–2.8: per-dataset tables detailing stratified sample counts for the datasets in Table 2.1. Section 3: Table 3.1 describes slide- and case-level sample counts for the biomarker prediction datasets (train/tune/test). Section 4 ‘Tile-level benchmarks’ describes additional metrics used to evaluate tile-level linear probing results. Table 4.1 describes the tile benchmark datasets. Table 4.2: tile-level results (additional metrics). Table 4.3: TCGA CRC-MSI tile-level biomarker prediction results (this task is not in the main figure). Table 4.4: PCAM and WILDS using a pretrained CNN from a breast-specialized model. Section 5 ‘Multitissue PanMSK dataset’ describes the preparation of the internal PanMSK tile-level benchmark dataset in detail. Table 5.1: PanMSK data splits. Fig. 5.1: tile distribution (tissues, cancer/benign). [file 41591_2024_3141_MOESM1_ESM.pdf]

nature medicine

Supplementary information https://doi.org/10.1038/s41591-024-03141-0

A foundation model for clinical-grade computational pathology and rare cancers detection

In the format provided by the authors and unedited

S Supplementary Notes

S.1 Early foundation models in computational pathology

Several computational pathology models have been released in the past couple of years. Wang et al. [1] introduced the first such model using data from The Cancer Genome Atlas (TCGA) [2] and the Pathology AI Platform (PAIP) [3] and a modified MoCoV3 [4] algorithm to train a 28M parameter Swin Transformer [5] model. Since then, several models using TCGA and different model architectures and training procedures have been released: Phikon [6] a ViT-B 86M parameter model using iBOT [7], Remedis [8] a ResNet-152 with 232M parameters, Ciga et al. [9] ResNets with 11-45M parameters using SIMCLR [10], and Lunit [11] a ViT-S 22M parameter models using DINO [12], MoCov2 [13], SwAV [14] and Barlow Twins [15]. UNI [16] and RudolphV [17] both leverage proprietary datasets of approximately 100k whole slide images (WSIs) to train a ViT-L 307M parameter model using DINOv2 [18]. Campanella et al. [19] also use a proprietary dataset of 400k WSIs, although they train a smaller ViT-S with 22M parameters using DINO [12] and MAE [20]. Several models have leveraged language data during training using CLIP [21]: PLIP using Twitter text [22], QUIILNet using YouTube audio and automatic speech recognition [23], and CONCH using PubMed [24]. There are two additional models which don't fit the broad categories above: HIPT [25] and LongViT [26]. HIPT employs a novel hierarchically trained architecture, hypothesized to better learn the inherent hierarchical structure of WSIs. LongViT is a slide-level foundation model based on a LongNet [27] architecture.

All of the aforementioned models are summarized in Supplementary Tab. S1.1.

Model	Data source	Data size		Model architecture	Model size	Objective function
		WSI	Tiles			
**Virchow**	MSKCC	1.5M	2B	ViT-H	632M	DINOv2
UNI [16]	Mass-100K	100K	100M	ViT-L	307M	DINOv2
RudolfV [17]	TCGA + Properitary	103K	750M	ViT-L	307M	DINOv2
Campanella et al. [19]	Mount Sinai	400K	3B	ViT-S	22M	DINO, MAE
Lunit [11]	TCGA + TULIP	37K	33M	ViT-S	22M	Various
Phikon [6]	TCGA	6K	43M	ViT-B	86M	iBOT
Remedis [8]	TCGA	29K	50M	ResNet-152	232M	SIMCLR
Ciga et al. [9]	TCGA + CPTAC ++	25K	4.2M	ResNet	11-45M	SIMCLR
CTransPath [1]	TCGA + PAIP	32K	15M	Swin Transformer	28M	MoCoV3
HIPT [25]	TCGA	11K	104M	ViT-HIPT	10M	DINO
LongViT [26]	TCGA	10K	1M	LongNet	22M	DINO
PLIP [22]	OpenPath	NA	200K	ViT-B*	86M	CLIP
QUILTNet [23]	Quilt-1M	NA	1M	ViT-B*	86M	CLIP
CONCH [24]	PMC-Path + EDU	NA	1.2M	ViT-B*	86M	CLIP

Supplementary Tab. S1.1: Summary of proposed foundation models in computational pathology highlighting the size of the training data, size of the model architecture, and training objective. The last three entries in the table combine vision and language data and train only using tiles. *The model architecture in these cases refers only to the tile embedding as opposed to the entire model size.

S.2 Clinical Evaluation

Dataset	Num Tissues	Ground Truth	Invasive	Non-Invasive	Total
Prostate product benchmark	1 (prostate)	block level	1991	956	2947
Prostate rare variants	1 (prostate)	slide level	28	112	140
Breast product benchmark	1 (breast)	slide level	190	1501	1691
Breast rare variants	1 (breast)	case level	98	392	490
BLN product benchmark	1 (lymph node)	slide level	458	295	753
LN rare variants	1 (lymph node)	specimen-level	48	192	240
Pan-tissue product benchmark	16 (see Tab. S2.2)	slide level	1145	1274	2419

Supplementary Tab. S2.1: Summary of datasets used in clinical validation.

Tissue	Invasive (slides)	Non-Invasive (slides)	Total (slides)
Bladder	69	75	144
Bone	73	39	112
Brain	26	16	42
Breast	55	47	102
Cervix	21	113	134
Colon	65	95	160
Endometrium	44	100	144
Liver	67	34	101
Lung	72	42	114
Lymph node	97	83	180
Pancreas	46	24	70
Peritoneum	80	80	160
Prostate	90	85	175
Skin	167	180	347
Stomach	82	86	168
Upper GI	82	173	255
Overall	1145	1274	2419

Supplementary Tab. S2.2: Number of slides for each stratum in pan-tissue product benchmark dataset.

Largest Tumor	Slides
Non-invasive	295
ITC ($\leq$ 0.2 mm)	47
Micrometastasis ($\geq$ 0.2 mm, $\leq$ 2 mm)	152
Macrometastasis ($\geq$ 2 mm)	259
Overall	753

Supplementary Tab. S2.3: Number of slides for each stratum in BLN product benchmark dataset.

Variant	Specimens
Non-invasive	192
DLBCL	26
FL	13
Hodgkin's lymphoma	2
MZL	5
Overall	240

Supplementary Tab. S2.4: Number of specimens for each stratum in LN rare variants dataset.

Stratum	Invasive (slides)	Non-Invasive (slides)	Total (slides)
Biopsy	61	225	286
Resection	128	1275	1403
IDC	143	1325	1468
ILC	45	1325	1370
Other invasive	4	1325	1329
Overall	190	1501	1691

Supplementary Tab. S2.5: Number of slides for each stratum in breast product benchmark dataset.

Variant	Cases
Non-invasive	389
IDC	11
ILC	11
Adenoid cystic carcinoma	9
Carcinoma with apocrine differentiation	9
Cribriform carcinoma	8
Invasive micropapillary carcinoma	8
Metaplastic carcinoma matrix producting subtype	3
Metaplastic carcinoma spindle cell	10
Metaplastic carcinoma squamous cell	5
Mucinous carcinoma	10
Secretory carcinoma	5
Tubular carcinoma	8
Overall	484

Supplementary Tab. S2.6: Number of cases for each stratum in breast rare variants dataset.

Stratum	Invasive (blocks)	Non-Invasive (blocks)	Total (blocks)
Tumor < 0.5 mm	197	956	1153
Tumor ≥ 0.5 mm	1731	956	2687
Overall	1991	956	2947

Supplementary Tab. S2.7: Number of blocks for each stratum in prostate product benchmark dataset.

Variant	Slides
Non-invasive	112
Atrophic	2
Foamy cell	10
Follicular lymphoma	3
Indefinite for lymphoma	1
Neuroendocrine	9
Small lymphocytic lymphoma	1
Overall	140

Supplementary Tab. S2.8: Number of slides for each stratum in prostate rare variants dataset.

S.3 Biomarker prediction from H&E

The training, validation, and testing distribution is shown in Supplementary Tab. S3.1 for each biomarker dataset.

Biomarker	Subset	Cases	Slides	PosProportion
Prostate-AR	train	1051	1461	0.18
	tune	348	480	0.20
	test	347	480	0.16
Ovarian-FGA	train	679	791	0.91
	tune	115	134	0.90
	test	111	126	0.88
Gastric-Her2	train	968	968	0.19
	tune	170	170	0.23
	test	161	161	0.17
Endometrial-PTEN	train	983	1038	0.48
	tune	164	170	0.43
	test	164	178	0.41
Skin-BRAF	train	782	868	0.25
	tune	131	137	0.21
	test	131	138	0.13
Colon-MSI	train	4609	11027	0.10
	tune	481	1417	0.14
	test	482	1446	0.14
Breast-CDH1	train	648	673	0.13
	tune	215	220	0.13
	test	214	228	0.13
Bladder-FGFR	train	520	542	0.24
	tune	259	275	0.29
	test	259	270	0.25
Lung-EGFR	train	2186	2858	0.28
	tune	356	457	0.29
	test	358	457	0.28

Supplementary Tab. S3.1: Statistics of the case-level biomarker target datasets, including the number of cases, the number of slides, and the proportion of positive labels.

S.4 Tile-level benchmarks

Further details about the input tiles for each of the tile-level benchmark tasks are shown in Supplementary Tab. S4.1.

Dataset	Tissue	High-Level Tissue Types	Classes	Res	Tile size	No. of tiles
PCam	Lymph node	1	2	10×	96×96	327,680
WILDS	Lymph node	1	2	10×	96×96	455,954
CRC	Colon	1	9	20×	224×224	107,180
CRC (no norm)	Colon	1	9	20×	224×224	107,180
PanMSK	PanCancer	17	2	20×	224×224	1,196,171
MHIST	Colon	1	2	5×	224×224	3,152
MIDOG	PanCancer	6	2	40×	224×224	21,806
TCGA TIL	PanCancer	12	2	20×	100×100	304,097
TCGA CRC-MSI	Colon	1	2	20×	512×512	51,918

Supplementary Tab. S4.1: Summary of the tile-level benchmark datasets used for linear probing.

Additional evaluation metrics for each model on the tile-level benchmarks are detailed in Supplementary Tab. S4.2. We report accuracy, balanced accuracy, and weighted F1 score. Balanced accuracy is calculated by averaging true positive rate (TPR) ($\text{TPR} = \frac{\text{TP}}{\text{TP+FN}}$) and true negative rate (TNR) ($\text{TNR} = \frac{\text{TN}}{\text{TN+FP}}$). Weighted F1 score is calculated by first calculating the F1 score (harmonic mean of precision and recall) for each class and then averaging the scores, weighted by the number of positive samples for each class. For balanced accuracy and weighted F1 score calculation, we use the probability threshold = 0.5 as the operating point.

Dataset	Metric	NatImg	PLIP	CTransPath	$\text{DINO}_{p=8}$	Phikon	Uni	Virchow
CRC	Accuracy	0.952 (0.947, 0.957)	0.946 (0.940, 0.951)	0.962 (0.958, 0.966)	0.959 (0.954, 0.963)	0.958 (0.953, 0.963)	0.962 (0.958, 0.966)	**0.973** (0.969, 0.976)
	Balanced Accuracy	0.926 (0.919, 0.933)	0.918 (0.911, 0.925)	0.947 (0.942, 0.953)	0.945 (0.939, 0.951)	0.944 (0.938, 0.950)	0.949 (0.943, 0.954)	**0.962** (0.956, 0.967)
	Weighted F1	0.952 (0.947, 0.957)	0.944 (0.938, 0.949)	0.962 (0.958, 0.966)	0.959 (0.955, 0.964)	0.959 (0.954, 0.963)	0.963 (0.958, 0.967)	**0.973** (0.969, 0.976)
WILDS	Accuracy	0.934 (0.932, 0.936)	0.870 (0.867, 0.872)	0.947 (0.945, 0.948)	0.958 (0.957, 0.959)	0.972 (0.971, 0.973)	**0.983** (0.982, 0.984)	0.971 (0.970, 0.972)
	Balanced Accuracy	0.934 (0.933, 0.936)	0.870 (0.867, 0.872)	0.947 (0.945, 0.948)	0.958 (0.957, 0.959)	0.972 (0.971, 0.973)	**0.983** (0.982, 0.984)	0.971 (0.970, 0.972)
	Weighted F1	0.934 (0.932, 0.936)	0.868 (0.865, 0.870)	0.947 (0.945, 0.948)	0.958 (0.957, 0.959)	0.972 (0.971, 0.973)	**0.983** (0.982, 0.984)	0.971 (0.970, 0.972)
TCGA TILs	Accuracy	0.931 (0.929, 0.934)	0.928 (0.925, 0.930)	0.933 (0.931, 0.935)	0.943 (0.941, 0.945)	0.945 (0.943, 0.947)	0.946 (0.944, 0.948)	**0.950** (0.948, 0.952)
	Balanced Accuracy	0.864 (0.860, 0.868)	0.859 (0.855, 0.864)	0.862 (0.858, 0.866)	0.880 (0.876, 0.884)	0.896 (0.892, 0.899)	0.895 (0.891, 0.899)	**0.905** (0.902, 0.909)
	Weighted F1	0.930 (0.927, 0.932)	0.926 (0.923, 0.928)	0.931 (0.929, 0.933)	0.942 (0.940, 0.944)	0.944 (0.943, 0.946)	0.945 (0.943, 0.947)	**0.949** (0.947, 0.951)
PanMSK	Accuracy	0.883 (0.882, 0.884)	0.862 (0.861, 0.864)	0.897 (0.896, 0.898)	0.902 (0.901, 0.904)	0.924 (0.922, 0.924)	0.943 (0.942, 0.944)	**0.950** (0.950, 0.951)
	Balanced Accuracy	0.883 (0.882, 0.884)	0.862 (0.861, 0.864)	0.897 (0.896, 0.898)	0.903 (0.901, 0.904)	0.924 (0.922, 0.925)	0.943 (0.942, 0.944)	**0.950** (0.950, 0.951)
	Weighted F1	0.883 (0.882, 0.884)	0.862 (0.861, 0.864)	0.897 (0.896, 0.898)	0.903 (0.901, 0.904)	0.923 (0.922, 0.924)	0.943 (0.942, 0.944)	**0.950** (0.950, 0.951)
CRC (no norm)	Accuracy	0.927 (0.921, 0.933)	0.793 (0.784, 0.803)	0.840 (0.831, 0.848)	0.949 (0.944, 0.954)	0.883 (0.876, 0.890)	0.941 (0.935, 0.946)	**0.968** (0.964, 0.972)
	Balanced Accuracy	0.895 (0.887, 0.903)	0.741 (0.731, 0.752)	0.825 (0.817, 0.833)	0.919 (0.911, 0.926)	0.872 (0.864, 0.880)	0.932 (0.925, 0.938)	**0.960** (0.955, 0.965)
	Weighted F1	0.928 (0.922, 0.933)	0.806 (0.796, 0.815)	0.844 (0.836, 0.852)	0.949 (0.944, 0.954)	0.888 (0.880, 0.895)	0.943 (0.938, 0.948)	**0.968** (0.964, 0.972)
PCam	Accuracy	0.887 (0.884, 0.891)	0.874 (0.871, 0.878)	0.872 (0.868, 0.875)	0.917 (0.914, 0.920)	0.905 (0.901, 0.908)	**0.934** (0.932, 0.937)	**0.933** (0.930, 0.936)
	Balanced Accuracy	0.887 (0.884, 0.890)	0.874 (0.871, 0.878)	0.872 (0.868, 0.875)	0.917 (0.914, 0.920)	0.905 (0.902, 0.908)	**0.934** (0.932, 0.937)	**0.933** (0.930, 0.936)
	Weighted F1	0.887 (0.883, 0.890)	0.874 (0.870, 0.877)	0.871 (0.868, 0.875)	0.917 (0.914, 0.920)	0.904 (0.901, 0.907)	**0.934** (0.932, 0.937)	**0.933** (0.930, 0.936)
MHIST	Accuracy	**0.831** (0.808, 0.855)	**0.799** (0.774, 0.824)	**0.818** (0.794, 0.842)	0.769 (0.742, 0.795)	**0.793** (0.769, 0.817)	**0.842** (0.818, 0.866)	**0.835** (0.812, 0.859)
	Balanced Accuracy	**0.826** (0.801, 0.853)	**0.784** (0.756, 0.811)	**0.802** (0.775, 0.828)	0.743 (0.713, 0.771)	**0.780** (0.753, 0.807)	**0.838** (0.813, 0.863)	**0.831** (0.805, 0.857)
	Weighted F1	**0.832** (0.808, 0.856)	**0.799** (0.773, 0.824)	**0.817** (0.794, 0.842)	0.766 (0.740, 0.793)	**0.794** (0.768, 0.817)	**0.843** (0.819, 0.866)	**0.836** (0.813, 0.860)
MIDOG	Accuracy	0.689 (0.676, 0.703)	0.638 (0.624, 0.652)	0.644 (0.630, 0.658)	0.678 (0.663, 0.692)	0.700 (0.687, 0.715)	0.749 (0.736, 0.761)	**0.787** (0.775, 0.799)
	Balanced Accuracy	0.689 (0.676, 0.702)	0.636 (0.623, 0.650)	0.643 (0.628, 0.657)	0.678 (0.663, 0.692)	0.699 (0.686, 0.714)	0.749 (0.736, 0.761)	**0.788** (0.775, 0.799)
	Weighted F1	0.689 (0.676, 0.703)	0.636 (0.622, 0.650)	0.643 (0.629, 0.657)	0.678 (0.663, 0.692)	0.700 (0.686, 0.714)	0.749 (0.736, 0.761)	**0.787** (0.775, 0.799)

Supplementary Tab. S4.2: Downstream task linear probing evaluations. Bolded values indicate the top scoring model for each task. More than one value is bolded when there is no statistically significant difference between the results ($p < 0.05$).

The linear probing evaluations on TCGA colorectal cancer (CRC)-microsatellite instability (MSI) data for Virchow, Uni, Phikon, and NatImg [18] (1.1B parameter model trained on 142 million natural images) are shown in Supplementary Tab. S4.3. Only those models that could take a 448×448 input tile were evaluated. While applying the linear probing protocol in Sec. 8.5.1 for TCGA-MSI produced a favourable result for Virchow, the result for Uni under-performed the one reported in [16]. A key difference to their approach was to not split out a validation set from the publicly provided training data. Doing so, allowed us to reproduce a similar result. It should be noted that Virchow, Phikon, and NatImg were not trained on tiles larger than 224×224, whereas Uni was fine-tuned on 512×512 tiles at 20× magnification.

We can see that Virchow embeddings outperform those of a specialist model on PCam and WILDS (Supplementary Tab. S4.4). The specialist model here is the tile embedding component extracted from a slide- or specimen-level cancer detection model for breast cancer metastases in lymph nodes (Paige Breast Lymph Node), trained in a weakly supervised manner with multiple instance learning (MIL).

Validation split?	Metric	NatImg	Phikon	Uni	Virchow
Yes	Accuracy	0.736 (0.732, 0.741)	0.752 (0.747, 0.757)	0.716 (0.711, 0.720)	**0.784** (0.779, 0.789)
	Balanced Accuracy	0.682 (0.677, 0.689)	**0.736** (0.729, 0.743)	0.693 (0.687, 0.701)	**0.736** (0.729, 0.744)
	Weighted F1	0.764 (0.760, 0.767)	0.780 (0.776, 0.783)	0.749 (0.746, 0.753)	**0.804** (0.800, 0.808)
No	Accuracy	0.752 (0.747, 0.755)	0.789 (0.784, 0.793)	**0.800** (0.795, 0.804)	**0.801** (0.796, 0.805)
	Balanced Accuracy	0.669 (0.664, 0.676)	0.699 (0.691, 0.706)	**0.719** (0.712, 0.724)	**0.733** (0.727, 0.741)
	Weighted F1	0.774 (0.769, 0.777)	0.803 (0.799, 0.807)	**0.813** (0.809, 0.817)	**0.816** (0.812, 0.819)

Supplementary Tab. S4.3: The linear probing evaluation results for TCGA CRC-MSI data, with two protocols: with 10% of the public training set split out into a validation set(our default protocol) and without a validation set. Numbers in bold highlight the statistically significantly ($p < 0.05$) top scoring results.

Dataset	Metric	BLN	Virchow
PCam	Accuracy	0.861	0.933
	Balanced Accuracy	0.861	0.933
	Weighted F1	0.860	0.933
WILDS	Accuracy	0.943	0.970
	Balanced Accuracy	0.943	0.970
	Weighted F1	0.942	0.970

Supplementary Tab. S4.4: Linear probing of embeddings from Virchow and the tile-embedder component of a weakly supervised (MIL) model specializing in cancer detection in breast lymph nodes (BLN).

S.5 Multi-tissue PanMSK dataset

Exhaustive annotations (i.e. a complete segmentation of cancer vs non-cancer regions across the entire WSI) were collected for 399 prostate slides, 187 breast slides, 115 bladder slides, 64 breast lymph node slides, and 55 colon slides by a different pathologist for each tissue group. For the other tissue groups (see Fig. 1d), a pathologist highlighted one or more cancer regions on each slide non-exhaustively. The fully-annotated 64 breast lymph node slides were combined with 48 lymph node slides with highlighted cancer regions, originating from various locations. We sampled non-cancer tiles from slides labeled as benign. With the exception of the endometrial tissue group (for which we selected cancer regions in 11 slides), no tissue group had less than 50 slides partially or thoroughly annotated.

PanMSK was split into training, validation, and testing subsets at the slide level (Supplementary Tab. S5.1), ensuring that no two subsets share tiles from the same slide. The number of cancer tiles per tissue group was capped at the median number of cancer tiles across all tissue groups. The subsets were balanced to achieve an approximately 7:1:2 ratio of both slides *and* tiles. The splits were determined algorithmically with the objective of keeping similar tissue type and label distributions cross splits, as shown in Supplementary Fig. S5.1. This objective was optimized iteratively. In each iteration, slides were randomly shuffled between the splits and a permutation was picked greedily to maximize the objective. After balancing cancer tiles across the training, validation, and testing subsets and across tissue groups, benign tiles were sampled per tissue group to achieve a 1:1 ratio between cancer and benign tiles.

Split	Slides	Cancer tiles	Benign tiles
Training	2,797	418,738	417,466
Validation	402	60,462	60,296
Testing	800	119,792	119,417

Supplementary Tab. S5.1: Slide and tile counts in the PanMSK dataset.

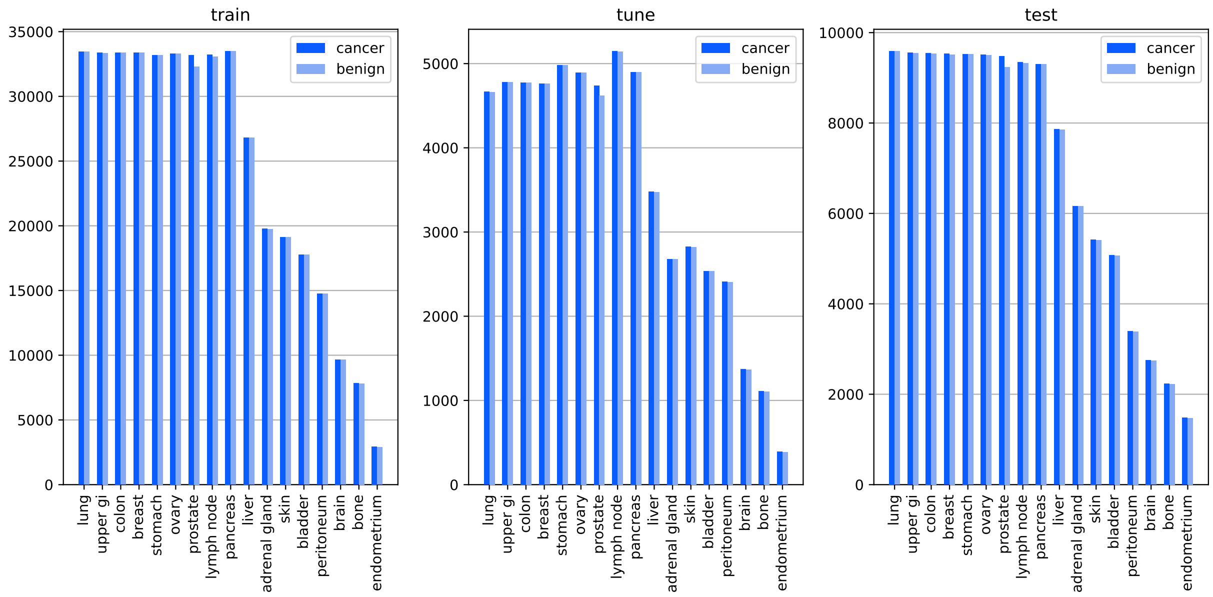

Supplementary Fig. S5.1: Distributions of cancer and benign tiles in the PanMSK dataset. The splits are balanced such that each tissue group approximately follows the same 7:1:2 (training:validation:testing) ratios in both tiles and slides counts.

References

[1] Xiyue Wang, Sen Yang, Jun Zhang, Minghui Wang, Jing Zhang, Wei Yang, Junzhou Huang, and Xiao Han. Transformer-based unsupervised contrastive learning for histopathological image classification. *Medical Image Analysis*, 81:102559, 2022.

[2] John N Weinstein, Eric A Collisson, Gordon B Mills, Kenna R Shaw, Brad A Ozenberger, Kyle Ellrott, Ilya Shmulevich, Chris Sander, and Joshua M Stuart. The cancer genome atlas pan-cancer analysis project. *Nature genetics*, 45(10):1113–1120, 2013.

[3] Yoo Jung Kim, Hyungjoon Jang, Kyoungbun Lee, Seongkeun Park, Sung-Gyu Min, Choyeon Hong, Jeong Hwan Park, Kanggeun Lee, Jisoo Kim, Wonjae Hong, et al. PAIP 2019: Liver cancer segmentation challenge. *Medical image analysis*, 67:101854, 2021.

[4] Xinlei Chen, Saining Xie, and Kaiming He. An empirical study of training self-supervised vision transformers. In *Proceedings of the IEEE/CVF International Conference on Computer Vision*, 2021.

[5] Ze Liu, Yutong Lin, Yue Cao, Han Hu, Yixuan Wei, Zheng Zhang, Stephen Lin, and Baining Guo. Swin transformer: Hierarchical vision transformer using shifted windows. In *Proceedings of the IEEE/CVF International Conference on Computer Vision*, 2021.

[6] Alexandre Filiot, Ridouane Ghermi, Antoine Olivier, Paul Jacob, Lucas Fidon, Alice Mac Kain, Charlie Saillard, and Jean-Baptiste Schiratti. Scaling self-supervised learning for histopathology with masked image modeling. Preprint at https://doi.org/10.1101/2023.07.21.23292757, 2023.

[7] Jinghao Zhou, Chen Wei, Huiyu Wang, Wei Shen, Cihang Xie, Alan Yuille, and Tao Kong. iBOT: Image bert pre-training with online tokenizer. Preprint at https://doi.org/10.48550/arXiv.2111.07832, 2021.

[8] Shekoofeh Azizi, Laura Culp, Jan Freyberg, Basil Mustafa, Sebastien Baur, Simon Kornblith, Ting Chen, Nenad Tomasev, Jovana Mitrović, Patricia Strachan, et al. Robust and data-efficient generalization of self-supervised machine learning for diagnostic imaging. *Nature Biomedical Engineering*, 7:1–24, 2023.

[9] Ozan Ciga, Tony Xu, and Anne Louise Martel. Self supervised contrastive learning for digital histopathology. *Machine Learning with Applications*, 7:100198, 2022.

[10] Ting Chen, Simon Kornblith, Mohammad Norouzi, and Geoffrey Hinton. A simple framework for contrastive learning of visual representations. In *Proceedings of the 37th International Conference on Machine Learning*, 2020.

[11] Mingu Kang, Heon Song, Seonwook Park, Donggeun Yoo, and Sérgio Pereira. Benchmarking self-supervised learning on diverse pathology datasets. In *Proceedings of the IEEE/CVF Conference on Computer Vision and Pattern Recognition*, 2023.

[12] Mathilde Caron, Hugo Touvron, Ishan Misra, Hervé Jégou, Julien Mairal, Piotr Bojanowski, and Armand Joulin. Emerging properties in self-supervised vision transformers. In *Proceedings of the IEEE/CVF International Conference on Computer Vision*, 2021.

[13] Xinlei Chen, Haoqi Fan, Ross Girshick, and Kaiming He. Improved baselines with momentum contrastive learning. Preprint at https://doi.org/10.48550/arXiv.2003.04297, 2020.

[14] Mathilde Caron, Ishan Misra, Julien Mairal, Priya Goyal, Piotr Bojanowski, and Armand Joulin. Unsupervised learning of visual features by contrasting cluster assignments. 2020.

[15] Jure Zbontar, Li Jing, Ishan Misra, Yann LeCun, and Stéphane Deny. Barlow twins: Self-supervised learning via redundancy reduction. In *Proceedings of the 38th International Conference on Machine Learning*, 2021.

[16] Richard J Chen, Tong Ding, Ming Y Lu, Drew FK Williamson, Guillaume Jaume, Andrew H Song, Bowen Chen, Andrew Zhang, Daniel Shao, Muhammad Shaban, et al. Towards a general-purpose foundation model for computational pathology. *Nature Medicine*, pages 1–13, 2024.

[17] Jonas Dippel, Barbara Feulner, Tobias Winterhoff, Simon Schallenberg, Gabriel Dernbach, Andreas Kunft, Stephan Tietz, Philipp Jurmeister, David Horst, Lukas Ruff, et al. RudolfV: A foundation model by pathologists for pathologists. Preprint at https://doi.org/10.48550/arXiv.2401.04079 , 2024.

[18] Maxime Oquab, Timothée Darcet, Théo Moutakanni, Huy Vo, Marc Szafraniec, Vasil Khalidov, Pierre Fernandez, Daniel Haziza, Francisco Massa, Alaaeldin El-Nouby, et al. DINOv2: Learning robust visual features without supervision. Preprint at https://doi.org/10.48550/arXiv.2304.07193, 2023.

[19] Gabriele Campanella, Ricky Kwan, Eugene Fluder, Jennifer Zeng, Aryeh Stock, Brandon Veremis, Alexandros D Polydorides, Cyrus Hedvat, Adam Schoenfeld, Chad Vanderbilt, et al. Computational pathology at health system scale–self-supervised foundation models from three billion images. Preprint at https://doi.org/10.48550/arXiv.2310.07033, 2023.

[20] Kaiming He, Xinlei Chen, Saining Xie, Yanghao Li, Piotr Dollár, and Ross Girshick. Masked autoencoders are scalable vision learners. In *Proceedings of the IEEE/CVF Conference on Computer Vision and Pattern Recognition*, 2022.

[21] Alec Radford, Jong Wook Kim, Chris Hallacy, Aditya Ramesh, Gabriel Goh, Sandhini Agarwal, Girish Sastry, Amanda Askell, Pamela Mishkin, Jack Clark, Gretchen Krueger, and Ilya Sutskever. Learning transferable visual models from natural language supervision. In *Proceedings of the 38th International Conference on Machine Learning*, 2021.

[22] Zhi Huang, Federico Bianchi, Mert Yuksekgonul, Thomas J Montine, and James Zou. A visual–language foundation model for pathology image analysis using medical twitter. *Nature medicine*, 29(9):2307–2316, 2023.

[23] Wisdom Oluchi Ikezogwo, Mehmet Saygin Seyfioglu, Fatemeh Ghezloo, Dylan Stefan Chan Geva, Fatwir Sheikh Mohammed, Pavan Kumar Anand, Ranjay Krishna, and Linda Shapiro. Quilt-1M: One million image-text pairs for histopathology. In *Advances in Neural Information Processing Systems*, 2023.

[24] Ming Y Lu, Bowen Chen, Drew FK Williamson, Richard J Chen, Ivy Liang, Tong Ding, Guillaume Jaume, Igor Odintsov, Long Phi Le, et al. A visual-language foundation model for computational pathology. *Nature Medicine*, 30:863–874, 2023.

[25] Richard J Chen, Chengkuan Chen, Yicong Li, Tiffany Y Chen, Andrew D Trister, Rahul G Krishnan, and Faisal Mahmood. Scaling vision transformers to gigapixel images via hierarchical self-supervised learning. In *Proceedings of the IEEE/CVF Conference on Computer Vision and Pattern Recognition*, 2022.

[26] Wenhui Wang, Shuming Ma, Hanwen Xu, Naoto Usuyama, Jiayu Ding, Hoifung Poon, and Furu Wei. When an image is worth 1,024 x 1,024 words: A case study in computational pathology. Preprint at https://doi.org/10.48550/arXiv.2312.03558, 2023.

[27] Jiayu Ding, Shuming Ma, Li Dong, Xingxing Zhang, Shaohan Huang, Wenhui Wang, Nanning Zheng, and Furu Wei. Longet: Scaling transformers to 1,000,000,000 tokens. Preprint at https://doi.org/10.48550/arXiv.2307.02486, 2023.
