# Supplementary material for: A foundation model for clinical-grade computational pathology and rare cancers detection
Source: Nat Med. 2024 Jul 22;30(10):2924–35. doi: 10.1038/s41591-024-03141-0 (PMC11485232; doi:10.1038/s41591-024-03141-0)
Supplement: Supplementary file 2 — Reporting Summary [file 41591_2024_3141_MOESM2_ESM.pdf]

nature portfolio

Corresponding author(s): Siqi Liu

Last updated by author(s): Jun 3, 2024

Reporting Summary

Nature Portfolio wishes to improve the reproducibility of the work that we publish. This form provides structure for consistency and transparency in reporting. For further information on Nature Portfolio policies, see our Editorial Policies and the Editorial Policy Checklist.

Statistics

For all statistical analyses, confirm that the following items are present in the figure legend, table legend, main text, or Methods section.

n/a	Confirmed	
☐	☒	The exact sample size (n) for each experimental group/condition, given as a discrete number and unit of measurement
☒	☐	A statement on whether measurements were taken from distinct samples or whether the same sample was measured repeatedly
☐	☒	The statistical test(s) used AND whether they are one- or two-sided<br>*Only common tests should be described solely by name; describe more complex techniques in the Methods section.*
☒	☐	A description of all covariates tested
☐	☒	A description of any assumptions or corrections, such as tests of normality and adjustment for multiple comparisons
☐	☒	A full description of the statistical parameters including central tendency (e.g. means) or other basic estimates (e.g. regression coefficient) AND variation (e.g. standard deviation) or associated estimates of uncertainty (e.g. confidence intervals)
☐	☒	For null hypothesis testing, the test statistic (e.g. F, t, r) with confidence intervals, effect sizes, degrees of freedom and P value noted<br>*Give P values as exact values whenever suitable.*
☒	☐	For Bayesian analysis, information on the choice of priors and Markov chain Monte Carlo settings
☒	☐	For hierarchical and complex designs, identification of the appropriate level for tests and full reporting of outcomes
☒	☐	Estimates of effect sizes (e.g. Cohen's d, Pearson's r), indicating how they were calculated

Our web collection on statistics for biologists contains articles on many of the points above.

Software and code

Policy information about availability of computer code

Data collection	For data collection, we used Python (3.10.11) along with Pandas (2.2.2) for indexing the data and metadata used for pretraining and benchmarking. OpenSlide (1.3.1) and Pillow (10.0.0) were used for preprocessing the image tiles for the benchmark. Where appropriate, we extracted per-specimen labels from clinical reports using DBT (1.5.0).
Data analysis	We used Python (3.10.11) for all experiments and analyses in the study, which can be replicated using open-source libraries as outlined below. For self-supervised pretraining, we used Pytorch (2.0.1) and Torchvision (0.15.1). The DINOv2 code was ported from the official repository (https://github.com/facebookresearch/dinov2) and adapted to Pytorch-lightning (1.9.0). All WSI processing during pretraining was performed online and was supported by cucim (23.10.0) and torchvision (0.16.1). For downstream task benchmarking, we use scikit-learn (1.4.2) for logistic regression and metrics computation. The baseline foundation models were obtained from the following links: Implementations of other visual pre-trained encoders benchmarked in the study are found at the following links: UNI (https://huggingface.co/MahmoodLab/UNI), Phikon (https://huggingface.co/owkin/phikon), DINOp=8 (https://github.com/lunit-io/benchmark-ssl-pathology), PLIP (https://huggingface.co/vinid/plip), CTransPath (https://github.com/Xiyue-Wang/TransPath) and the original natural image pre-trained DINOv2 (https://github.com/facebookresearch/dinov2).

For manuscripts utilizing custom algorithms or software that are central to the research but not yet described in published literature, software must be made available to editors and reviewers. We strongly encourage code deposition in a community repository (e.g. GitHub). See the Nature Portfolio guidelines for submitting code & software for further information.

Data

Policy information about availability of data

All manuscripts must include a data availability statement. This statement should provide the following information, where applicable:

- Accession codes, unique identifiers, or web links for publicly available datasets
- A description of any restrictions on data availability
- For clinical datasets or third party data, please ensure that the statement adheres to our policy

This study did not specifically collect patient data. The retrospective analysis utilized proprietary de-identified digital pathology whole slides and associated metadata exclusively licensed by Paige.AI, Inc. from Memorial Sloan Kettering Cancer Center (MSKCC). Requests for data need to be submitted to Paige AI (https://paige.ai/contact-us/) and evaluated by Paige AI and MSKCC on a case-by-case basis. All requests complying with internal regulations on data privacy and intellectual property will be granted. This study also utilized the following publicly available datasets for downstream benchmarking: CRC (NCT-CRC-HE-100K and NCT-CRC-HE-100K-NONORM; https://zenodo.org/records/1214456), WILDS (Camelyon17; https://wilds.stanford.edu/get_started), PCam (https://github.com/basveeling/pcam), MHIST (https://bmirds.github.io/MHIST), TCGA TIL (https://zenodo.org/records/6604094), MIDOG (https://midog.deepmicroscopy.org/download-dataset/), TCGA CRC-MSI (https://zenodo.org/records/3832231).

Human research participants

Policy information about studies involving human research participants and Sex and Gender in Research.

Reporting on sex and gender	Gender or sex was not included as a covariate at any stage of our experimental analysis.
Population characteristics	We did not collect or use any covariates pertaining to population characteristics at any stage of the study.
Recruitment	No patient recruitment was necessary for using histology whole slide images retrospectively.
Ethics oversight	Institutional review board (IRB) review was not applicable for the research described in this study. This research study was conducted retrospectively from de-identified data licensed to Paige.AI, Inc. from Memorial Sloan Kettering Cancer Center (MSKCC). The data used in this study were all collected originally for clinical use by MSKCC in the practice setting and are therefore considered secondary data. Only data previously de-identified by MSKCC were utilized in the analysis, and unique patient identifiers were completely removed from the analytical dataset.

Note that full information on the approval of the study protocol must also be provided in the manuscript.

Field-specific reporting

Please select the one below that is the best fit for your research. If you are not sure, read the appropriate sections before making your selection.

☒ Life sciences ☐ Behavioural & social sciences ☐ Ecological, evolutionary & environmental sciences

For a reference copy of the document with all sections, see nature.com/documents/nr-reporting-summary-flat.pdf

Life sciences study design

All studies must disclose on these points even when the disclosure is negative.

Sample size	No sample size calculations were conducted.<br>A total of 1,488,550 H&E whole histopathology slide images were gathered for training the foundation model. The superior performance of our pretrained model compared to all other baselines indicates that the sample size was sufficient. For information on downstream datasets, please refer to the datasets and evaluation subsection in the Methods section of the manuscript.
Data exclusions	No particular data exclusion was performed.
Replication	Attempts at replication were successful for the reported model results. The open-sourced model can be applied at https://huggingface.co/paige-ai/Virchow. The SDK for replicating the aggregator experiments can be accessed at https://github.com/Paige-AI/paige-ml-sdk
Randomization	For downstream evaluation involving the creation of training, validation, and test splits, we utilized the official splits provided by the original investigators of each dataset whenever available. When such splits were not available, we created them randomly. Generally, we created random splits stratified by class, ensuring similar class proportions across splits, and, if possible, at the patient level, ensuring that slides from the same patient were kept within the same split. The random seeds were fixed, and the splits were documented to ensure replicability.
Blinding	Blinding is not necessary for our study.

Reporting for specific materials, systems and methods

We require information from authors about some types of materials, experimental systems and methods used in many studies. Here, indicate whether each material, system or method listed is relevant to your study. If you are not sure if a list item applies to your research, read the appropriate section before selecting a response.

Materials & experimental systems

n/a	Involved in the study
☒	☐ Antibodies
☒	☐ Eukaryotic cell lines
☒	☐ Palaeontology and archaeology
☒	☐ Animals and other organisms
☒	☐ Clinical data
☒	☐ Dual use research of concern

Methods

n/a	Involved in the study
☒	☐ ChIP-seq
☒	☐ Flow cytometry
☒	☐ MRI-based neuroimaging
